# Global Trends, Inequalities, and Citation Dynamics in Burnout Research Among Healthcare Professionals: A Bibliometric Analysis (1987–2024)

**DOI:** 10.3390/healthcare14152356

**Published:** 2026-08-02

**Authors:** Elena Donisa, Solange Tamara Roșu, Vasile Eduard Roșu, Elena Mihaela Cărăușu

**Affiliations:** 1 Department of Medical Specialties II, Discipline of Nursing, Faculty of Medicine, “Grigore T. Popa” University of Medicine and Pharmacy, 700115 Iasi, Romania; elena.bejenariu-donisa@d.umfiasi.ro; 2 Department of Mother and Child Medicine, Discipline of Pediatrics, Faculty of Medicine, “Grigore T. Popa” University of Medicine and Pharmacy, 700115 Iasi, Romania; eduard.rosu@umfiasi.ro; 3 Faculty of Medicine, “Grigore T. Popa” University of Medicine and Pharmacy, 700115 Iasi, Romania; elena.carausu@umfiasi.ro

**Keywords:** burnout, healthcare professionals, bibliometric analysis, science mapping, citation analysis, multilevel citation modelling, workforce sustainability, organizational resilience

## Abstract

**Background/Objectives:** Burnout among healthcare professionals has emerged as a major occupational and organizational challenge with important implications for workforce sustainability, patient safety, and healthcare system performance. Although the scientific literature on burnout has expanded substantially, an integrated understanding of its global development, research structure, and scientific impact remains limited. This study aimed to examine the evolution, thematic development, and citation dynamics of burnout research among healthcare professionals through an integrated bibliometric approach combining science mapping, multilevel citation modelling, and critical literature synthesis. **Methods:** A bibliometric analysis was conducted using publications indexed in the Web of Science Core Collection between 1987 and 2024. Science mapping techniques were applied using Bibliometrix and VOSviewer to evaluate publication trends, collaboration networks, thematic development, and citation patterns. In addition, a multilevel negative binomial regression model was used to identify publication characteristics associated with citation impact. **Results:** A total of 1232 publications were included in the analysis. Scientific production increased markedly after 2020, temporally coinciding with the COVID-19 pandemic and growing scientific interest in healthcare workforce wellbeing. Research output was concentrated in high-income countries, particularly the United States, China, the United Kingdom, Canada, and Australia. Physicians and nurses dominated the literature, while non-clinical healthcare workers remained underrepresented. The thematic analysis indicated an increasing emphasis on organizational and system-level determinants of burnout alongside the continued presence of individual-centered perspectives. The multilevel negative binomial model identified longer title length and a greater number of author-provided keywords as being associated with lower expected citation counts, whereas a greater number of Keywords Plus terms was associated with higher expected citation counts. **Conclusions**: Burnout research has evolved into a rapidly expanding and increasingly interdisciplinary field. However, important gaps persist regarding geographic representation, workforce diversity, methodological standardization, and organizational intervention research. Future studies should move beyond descriptive approaches and further evaluate organizational interventions and workforce-related strategies to strengthen the evidence base that may inform healthcare policy and organizational practice.

## 1. Introduction

Burnout among healthcare professionals has emerged as one of the most significant occupational health challenges threatening the sustainability of contemporary healthcare systems. Over the past four decades, growing concerns regarding workforce wellbeing, staff retention, patient safety, and healthcare quality have transformed burnout from a relatively specialized psychological construct into a central topic in health services research, occupational medicine, nursing science, and healthcare management. The consequences of burnout extend far beyond individual psychological distress, contributing to workforce shortages, reduced professional performance, increased absenteeism, staff turnover, diminished quality of care, and compromised patient safety [1,2,3,4].

The importance of burnout has become particularly evident in the context of persistent healthcare workforce shortages, population ageing, increasing service demands, and growing organizational complexity. Compared with many other occupational groups, healthcare professionals operate in environments characterized by continuous exposure to high workloads, emotional demands, administrative pressures, shift work, ethical dilemmas, and chronic resource constraints. These working conditions not only increase the risk of emotional exhaustion, depersonalization, disengagement, and reduced professional accomplishment but may also compromise workforce retention, continuity of care, and the overall resilience of healthcare systems [4,5,6].

Although burnout was initially conceptualized primarily as an individual psychological response to prolonged occupational stress, this perspective has evolved considerably over the past decade. Contemporary evidence increasingly supports a systems-oriented approach, recognizing burnout as the result of complex interactions between individual, organizational, and health system factors. Organizational structures, staffing adequacy, leadership quality, workplace culture, administrative burden, and broader health policy environments are now recognized as key determinants shaping both the development and persistence of burnout among healthcare professionals. Today, burnout is widely regarded as both an individual and organizational phenomenon, reflecting the dynamic interaction between personal vulnerability and structural workplace conditions [1,4,6,7].

This systems-oriented perspective became particularly prominent during the COVID-19 pandemic, a period that coincided with increased scientific and public interest in burnout among healthcare professionals. Healthcare workers faced unprecedented clinical demands, risks of infection, rapidly changing protocols, shortages of personal protective equipment, moral distress, and prolonged exposure to severe illness and death. Coinciding with the COVID-19 pandemic, burnout research expanded rapidly across multiple disciplines, generating a substantial body of literature examining prevalence, risk factors, psychological consequences, organizational determinants, and intervention strategies. During this period, research priorities increasingly emphasized workforce wellbeing, organizational resilience, and the critical role of healthcare systems in protecting their workforce during periods of crisis [4,6,7,8].

Despite the substantial expansion of burnout research, important gaps continue to limit a comprehensive understanding of the field. First, considerable heterogeneity persists in the conceptualization and measurement of burnout, with studies employing different assessment instruments and operational definitions that hinder direct comparisons across findings [9,10,11]. Second, the available evidence remains disproportionately focused on physicians and nurses, whereas allied health professionals, administrative personnel, support staff, and other essential healthcare workforce groups remain comparatively underrepresented. Third, significant geographic inequalities characterize the evidence base, with most indexed publications originating from high-income countries despite the likelihood that occupational burnout poses equally important challenges in lower-resource healthcare systems. Collectively, these limitations have resulted in an incomplete and uneven understanding of the global development of burnout research among healthcare professionals [4,5,6].

Several systematic reviews and meta-analyses have synthesized the expanding literature on burnout among healthcare professionals, providing valuable evidence on prevalence estimates, risk factors, intervention effectiveness, and differences across professional groups [10,11,12,13,14]. However, most of these studies have focused on specific professions, limited geographical regions, particular clinical specialties, or narrowly defined research questions. Consequently, they provide only a partial understanding of how burnout research has evolved as a global scientific field. Critical aspects—including patterns of scientific production, thematic evolution, international collaboration, citation dynamics, and structural inequalities in research representation—remain insufficiently explored. Addressing these knowledge gaps requires bibliometric approaches capable of examining not only the findings of burnout research but also the evolution, structure, and scientific impact of the field itself [15,16,17,18].

Bibliometric approaches provide a powerful framework for addressing these knowledge gaps by examining the evolution of scientific knowledge itself. Unlike conventional systematic reviews or meta-analyses, bibliometric analyses enable the investigation of publication growth, collaboration networks, thematic evolution, geographic disparities, and patterns of scientific visibility within an integrated analytical framework. When complemented by a critical literature synthesis, these methods offer insights not only into what is currently known about burnout, but also into how scientific knowledge has evolved, been disseminated, and shaped across different healthcare systems, professional groups, and research communities, thereby facilitating the identification of emerging research priorities, underrepresented populations, and structural gaps that may not be apparent through conventional evidence syntheses alone [16,17,18].

Building upon previous bibliometric investigations, the present study provides a broader and more integrated assessment of burnout research among healthcare professionals by simultaneously examining publication trends, thematic evolution, geographic inequalities, and citation dynamics within a unified analytical framework [15,19,20,21]. Unlike previous bibliometric studies that primarily focused on descriptive indicators of scientific production, this study combines science mapping techniques with a critical literature synthesis and multilevel citation modelling to explore both the development and the scientific visibility of burnout research [16,17,18]. By integrating these complementary methodological approaches, the study provides a comprehensive overview of global burnout research published between 1987 and 2024, with particular emphasis on patterns of scientific growth, international collaboration, thematic evolution, underrepresented populations, structural gaps in the evidence base, and factors associated with research visibility [15,16,17,18,19,20,21].

Despite the growing number of bibliometric studies on burnout, existing analyses remain fragmented, frequently focusing on specific professional groups or isolated aspects of scientific production while relying predominantly on descriptive bibliometric indicators such as publication volume, citation counts, and keyword frequencies. Consequently, an integrated understanding of burnout research—including its thematic evolution, geographic inequalities, collaboration patterns, citation dynamics, and determinants of scientific visibility—remains limited, as comparatively little attention has been devoted to integrating bibliometric mapping, critical literature synthesis, and multilevel citation modelling within a unified analytical framework. By combining these complementary approaches, the present study provides a more comprehensive understanding of how burnout research has evolved, which areas have attracted the greatest scientific attention, and where important knowledge gaps continue to persist [15,19,20,21]. To further justify the need for the present bibliometric investigation, it is important to consider the main conclusions and limitations of existing systematic reviews and meta-analyses on burnout among healthcare professionals.

Over the past decade, an increasing number of systematic reviews and meta-analyses have examined the rapidly expanding literature on burnout among healthcare professionals [19,20,21]. These reviews have substantially improved understanding of prevalence patterns, risk factors, intervention effectiveness, and profession-specific differences. However, they also reveal important methodological limitations and persistent knowledge gaps that continue to affect the development of the field [19,20,21].

The reviews discussed in this section were selected because of their methodological influence, citation impact, and relevance to major conceptual developments in burnout research.

One of the earliest influential syntheses was conducted by West et al., who evaluated interventions aimed at reducing physician burnout. Their analysis suggested that both individual-level and organizational interventions can achieve modest reductions in burnout symptoms. Strategies such as workload reduction, enhanced teamwork, mindfulness training, and organizational support demonstrated beneficial effects. Nevertheless, most included studies reported only short-term outcomes, limiting conclusions regarding the long-term sustainability of intervention effects [12].

Building on this evidence, Panagioti et al. conducted a meta-analysis of controlled intervention studies and reported that organizational interventions tended to produce larger effects than individual-focused approaches. Their work reinforced the growing recognition that burnout cannot be effectively addressed solely through individual coping strategies. However, considerable heterogeneity among interventions, outcome measures, and burnout definitions complicated direct comparisons across studies and reduced the generalizability of findings [13].

A major methodological concern was highlighted by Rotenstein et al., who demonstrated substantial variation in reported burnout prevalence among physicians. Reported prevalence estimates ranged from 0% to 80.5%, depending largely on the instruments, cut-off values, and operational definitions employed. This analysis highlighted one of the most persistent challenges in burnout research: the absence of universally accepted measurement standards. As a result, comparisons between studies, healthcare systems, and professional groups remain problematic [10].

Subsequent systematic reviews expanded the focus beyond physicians. Woo et al. synthesized evidence regarding burnout among nurses and identified excessive workload, staffing shortages, emotional labor, and workplace stress as major contributors. Their findings suggested that burnout determinants differ considerably across professional groups and that intervention strategies should be tailored to the specific characteristics of different healthcare occupations. However, most available studies remained cross-sectional, limiting understanding of causal pathways and long-term trajectories [11].

The COVID-19 pandemic prompted a new wave of research examining burnout under crisis conditions. Reviews conducted by Ghahramani et al. and Leo et al. consistently reported increased levels of emotional exhaustion, anxiety, psychological distress, and occupational strain among healthcare workers. Common risk factors included fear of infection, inadequate protective equipment, increased workload, and exposure to patient mortality. While these studies provided valuable insight into pandemic-related stressors, many relied on rapid data collection methods and self-reported measures, raising concerns regarding methodological consistency and comparability [6,7].

More recent syntheses have attempted to integrate findings across broader segments of the healthcare workforce. Li et al. identified continuing challenges related to inconsistent definitions, variable measurement instruments, and differences in study design. Their findings also underscored the limited availability of longitudinal evidence, making it difficult to understand how burnout develops, persists, or resolves throughout professional careers [14].

Another important observation emerging from the literature concerns the uneven representation of healthcare occupations. While physicians and nurses account for the majority of published studies, comparatively little attention has been directed toward administrative personnel, allied health professionals, support staff, and other non-clinical workforce groups. Existing evidence suggests that these populations may experience distinct occupational stressors and organizational pressures that remain insufficiently investigated [4,5].

Taken together, existing studies have substantially advanced knowledge regarding burnout among healthcare professionals. Nevertheless, several limitations remain remarkably consistent across the literature. These include methodological heterogeneity, limited longitudinal evidence, insufficient representation of non-clinical healthcare workers, underrepresentation of lower-resource settings, and a continued emphasis on problem description rather than evaluation of organizational and system-level solutions [4,10,11,12,13].

These persistent limitations provide a strong rationale for a broader examination of the field. Rather than focusing exclusively on prevalence estimates or intervention outcomes, there is a need to explore how burnout research has evolved over time, which themes have attracted the greatest scholarly attention, which professional groups and geographic regions remain underrepresented, and which factors influence the visibility and scientific impact of published evidence. To address these gaps, the present study combines bibliometric methods with a critical literature synthesis of the global burnout literature, offering a comprehensive overview of research trends, knowledge structures, and emerging priorities in the field [15,19,20,21].

Accordingly, a comprehensive assessment of publication patterns, thematic evolution, geographic inequalities, collaboration networks, and citation dynamics is needed to achieve a more integrated understanding of how burnout research among healthcare professionals has evolved over time. Such an assessment may not only clarify the scientific development of the field but also identify persistent evidence gaps, underrepresented populations, and emerging research priorities relevant to workforce planning, organizational resilience, healthcare policy, and the long-term sustainability of healthcare systems [15,19,20,21].

To better position the present study within the existing literature, Table 1 summarizes the main characteristics of representative bibliometric studies, including recent investigations extending the analysis to the broader healthcare workforce [22], and highlights the methodological advances introduced by the present investigation.

As shown in Table 1, previous bibliometric studies have primarily concentrated on profession-specific populations, particularly nurses, and have mainly employed descriptive bibliometric approaches. In contrast, the present study extends the existing literature by examining the entire healthcare workforce and integrating science mapping, thematic evolution, geographic inequalities, citation dynamics, and multilevel citation modelling within a unified analytical framework. This comprehensive approach constitutes one of the principal scientific contributions of the present study, enabling a broader understanding of the evolution, visibility, and structural gaps of burnout research among healthcare professionals while providing evidence to guide future research priorities and healthcare workforce policy [19,20,21].

The present exploratory bibliometric study was designed to address the following research objectives, which collectively address the principal research questions concerning the evolution, thematic structure, geographic distribution, citation dynamics, and knowledge gaps in burnout research among healthcare professionals.

The study pursued the following objectives:

1. To examine the evolution of scientific production on burnout among healthcare professionals between 1987 and 2024.
2. To identify the leading journals, countries, institutions, authors, and international collaboration networks contributing to burnout research.
3. To analyse the thematic evolution and intellectual structure of the field using science mapping techniques.
4. To explore geographic and professional inequalities in research representation across the global healthcare workforce.
5. To evaluate publication characteristics associated with citation impact using multilevel citation modelling.
6. To identify persistent knowledge gaps and future research priorities relevant to future research, workforce management, and organizational practice.

## 2. Materials and Methods

### 2.1. Selecting Data Sources

The Web of Science (WoS) Core Collection was used as the sole bibliographic database for literature retrieval. It was selected because it provides a standardized and high-quality bibliographic dataset, consistent citation indexing, and broad compatibility with widely used bibliometric software, including the Bibliometrix package in R (R Foundation for Statistical Computing, Vienna, Austria) and VOSviewer (Centre for Science and Technology Studies (CWTS), Leiden University, Leiden, The Netherlands). These characteristics support science mapping, citation-based analyses, and the reproducibility of bibliometric investigations. Although Scopus, PubMed, Embase, CINAHL, PsycINFO, Google Scholar, and regional bibliographic databases represent valuable alternative sources of scientific literature, each differs in journal coverage, indexing policies, citation processing, and metadata standardization. Therefore, the selection of the Web of Science Core Collection reflected methodological consistency and suitability for the objectives of the present study rather than an assumption of database superiority [16,17,18].

Additionally, the Web of Science Core Collection enables comprehensive analyses across multiple dimensions, including institutional affiliations, geographic distribution, citation networks, and subject classifications. These features support the investigation of the interdisciplinary nature and global evolution of burnout research among healthcare professionals. By focusing on literature published between 1987 and 2024 and restricting the dataset to peer-reviewed journal articles published in English, the study ensured methodological consistency and alignment with its research objectives [16,17,18].

### 2.2. Inclusion and Exclusion Criteria

We applied inclusion and exclusion criteria during the data selection process to ensure the data were relevant, of high quality, and consistent for bibliometric analysis. Articles published from 1987 to 2024 were included to capture nearly four decades of research trends, including the period encompassing the COVID-19 pandemic. Only peer-reviewed journal articles and review articles published in English were included to ensure methodological consistency and the reliability of the bibliometric analyses. Included articles were required to explicitly address occupational burnout in healthcare human resources, including studies involving physicians, nurses, allied health professionals, and healthcare administrative personnel [15].

We excluded editorials, letters, book chapters, conference abstracts, theses, and commentaries due to their limited empirical contribution and lack of citation stability. Articles focusing on burnout in non-healthcare sectors (e.g., education, corporate, or IT industries) were excluded unless they analyzed healthcare-specific subgroups. Titles and abstracts were independently screened by two investigators to verify that the retrieved records addressed occupational burnout among healthcare professionals while excluding publications in which the term “burnout” referred to unrelated contexts (e.g., mechanical burnout or energy systems), according to the predefined eligibility criteria. Any discrepancies regarding study eligibility were resolved through discussion until consensus was reached. We identified and removed duplicate records or multiple entries of the same study across sub-databases to avoid redundancy. To ensure the robustness of the critical literature synthesis, publications identified as retracted were not considered when interpreting the evidence and research trends. The bibliographic dataset contained one retracted publication identified in the Web of Science records. This publication was retained in the descriptive bibliometric database for transparency purposes but was excluded from the critical interpretation of the evidence and from the critical literature synthesis. This filtering ensured that the final dataset was focused, methodologically consistent, and appropriate for examining research trends, knowledge gaps, and citation patterns related to occupational burnout among healthcare professionals [15].

### 2.3. Search Strategy for Articles Relevant to the Analysis

The search strategy combined terms related to burnout, occupational stress, healthcare professionals, mental health, and workload. Broad umbrella terms describing healthcare professionals (e.g., “healthcare workers”, “medical staff”, and “health professionals”) were intentionally selected to maximize the retrieval of publications representing diverse healthcare occupations rather than restricting the search to specific professional groups. Keywords and Boolean operators were iteratively refined through pilot searches to maximize sensitivity, specificity, and relevance. These umbrella descriptors were intended to capture publications concerning physicians, nurses, pharmacists, dentists, allied health professionals, paramedics, therapists, technicians, healthcare assistants, managers, administrative personnel, and other healthcare workforce groups indexed under these broader terms [4,5,6,10,11,12,13,14].

The final literature search was conducted on 11 April 2025 using the Web of Science Core Collection database. The search was performed in the Topic (TS) field of the Web of Science Core Collection using the complete Boolean query presented below. No additional database-specific restrictions beyond the predefined document type, language, and timespan criteria were applied. The search covered the period from 1987 to 2024, and the document type and language filters were subsequently applied according to the predefined inclusion and exclusion criteria described in Section 2.2.

The complete Boolean search query used to retrieve the bibliometric dataset is reported below:

*(“burnout” OR “occupational stress” OR “job fatigue”) AND (“healthcare workers” OR “medical staff” OR “health professionals”) AND (“mental health” OR “psychological wellbeing” OR “workload”) NOT (“students” OR “educators”)*

[15]

Preliminary searches were conducted to estimate the volume of available literature and to refine the search strategy where necessary. The strategy was iteratively refined through pilot searches and assessed for content validity by verifying its ability to retrieve landmark publications and major evidence syntheses in the field, including key systematic reviews and meta-analyses on burnout among healthcare professionals [10,11,12,13,14]. Although alternative search expressions (e.g., job burnout, professional burnout, occupational burnout, and emotional exhaustion) may identify additional records, the final search strategy was selected to provide a consistent and reproducible dataset aligned with the objectives of the present bibliometric analysis [10,11,12,13,14]. The ability of the strategy to retrieve landmark publications and major evidence syntheses in the field was verified during the pilot-search phase. The final dataset consisted of 1232 documents retrieved from the Web of Science Core Collection (Table 2).

Because the Web of Science Core Collection assigns multiple document-type labels to certain records, the final dataset included a small number of publications simultaneously indexed as “Article; Proceedings Paper” or “Article; Book Chapter”. These records were retained because they were indexed as journal articles and fulfilled all predefined eligibility criteria. The complete search strategy, including the search field, Boolean syntax, search date, timespan, and eligibility criteria, is reported to facilitate the reproducibility of the bibliographic dataset [15].

After identifying the relevant articles, we systematically extracted data. Key information extracted included the article title, author names, year of publication, journal name, study design, methodology, and key findings related to burnout. Reference management software (EndNote 21, Clarivate, Philadelphia, PA, USA) was used to organize records and facilitate data extraction. We conducted a bibliometric analysis of the extracted data using the Bibliometrix package in R (R Foundation for Statistical Computing, Vienna, Austria) [17]. Bibliometric software such as Bibliometrix and VOSviewer (Centre for Science and Technology Studies (CWTS), Leiden University, Leiden, The Netherlands) facilitates the visualization of citation networks, co-authorship patterns, and keyword co-occurrences, thereby supporting the exploration of the intellectual structure of burnout research among healthcare professionals [17,18].

### 2.4. Data Analysis Methods

**Figure 1:**
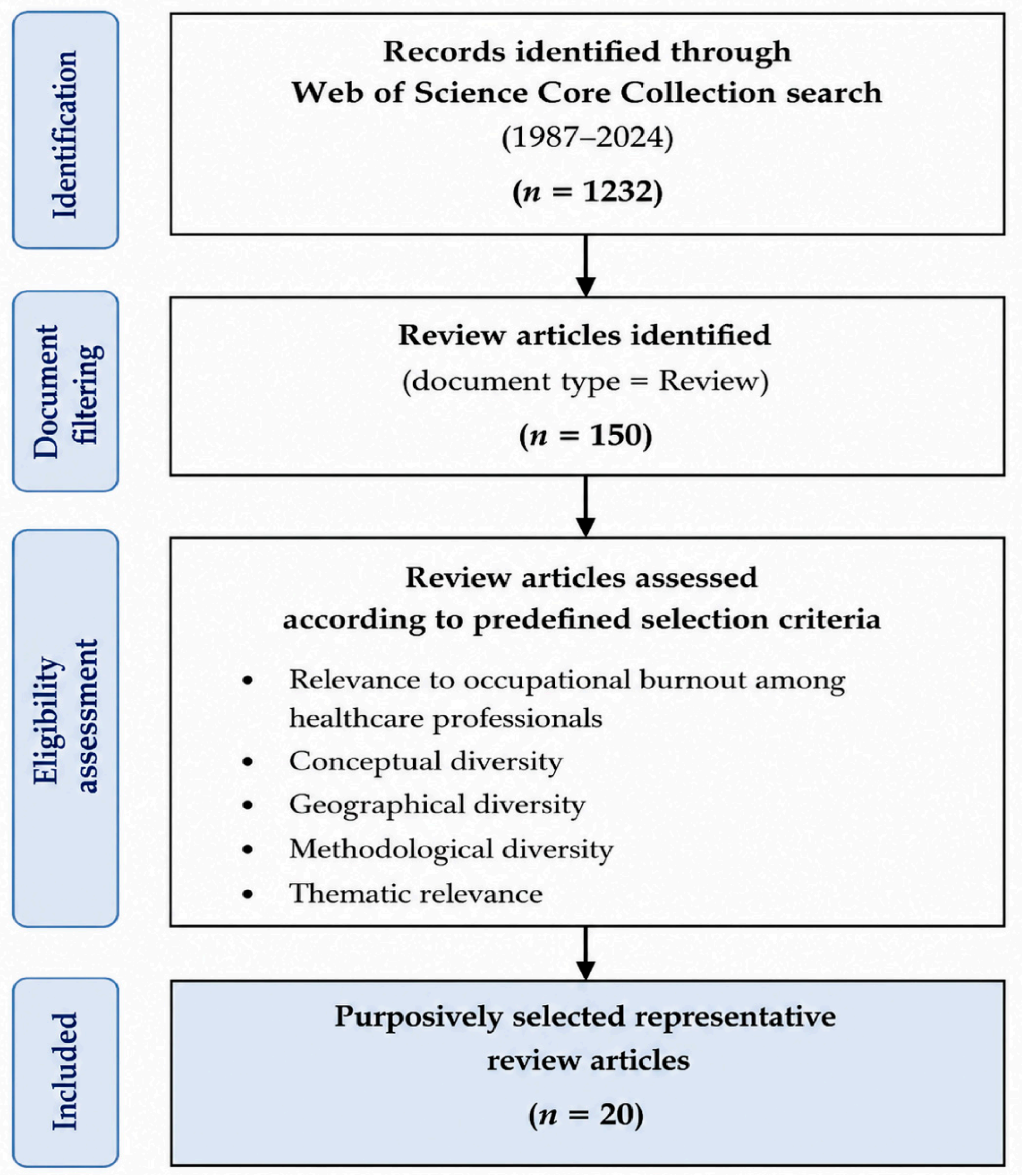
PRISMA-style flow diagram illustrating the selection process of the 20 representative review articles included in the structured critical literature synthesis from the 150 review articles identified in the bibliometric dataset.

After exporting and processing the bibliographic records retrieved from the Web of Science Core Collection, we conducted descriptive and exploratory analyses to assess annual publication trends, journal distribution, country and institutional contributions, and the frequency of keywords and subject categories. Bibliometric analyses were conducted in R using the Bibliometrix package, whereas VOSviewer was used for network visualization. Bibliometrix was used to generate the temporal evolution analysis and thematic map, while VOSviewer was used to construct the keyword co-occurrence network. Methodological details required for the interpretation of each bibliometric visualization are provided in the corresponding figure captions. The temporal evolution analysis illustrates the chronological appearance and persistence of the most frequently occurring terms across the study period (1987–2024), allowing visualization of thematic changes over time [17,18]. In the keyword co-occurrence network, nodes represented individual keywords, node size reflected keyword occurrence frequency, and links represented keyword co-occurrence relationships between terms. Link thickness reflected the strength of co-occurrence between two keywords. The thematic map was generated based on the centrality and density of the identified themes, allowing their classification into motor themes, niche themes, basic themes, and emerging or declining themes according to their conceptual development and relevance within the research field. Author Keywords and Keywords Plus were analysed as distinct bibliographic metadata fields according to the objectives of each bibliometric analysis and were not automatically merged into a single keyword dataset. Before interpreting the keyword networks, synonymous expressions, spelling variants, singular and plural forms, abbreviations, and equivalent terms were reviewed and standardized where appropriate to reduce semantic fragmentation and improve the consistency of the thematic analyses. Residual diagnostics for the multilevel regression models were evaluated using the DHARMa package in R (R Foundation for Statistical Computing, Vienna, Austria) [17,18]. A structured critical literature synthesis was conducted on a purposively selected subset of 20 representative review articles to complement the bibliometric analyses and provide a deeper contextual interpretation of research gaps, neglected populations, methodological limitations, and emerging directions in occupational burnout research among healthcare professionals. To improve methodological transparency, the study selection process is summarized in a PRISMA-style flow diagram (Figure 1). From the 150 review articles identified within the bibliometric dataset, studies were assessed according to predefined selection criteria, including relevance to occupational burnout among healthcare professionals, conceptual and methodological diversity, geographical representation, and thematic relevance. The eligibility of the review articles was independently assessed by two investigators according to the predefined selection criteria, and any differences in study selection were resolved through discussion until consensus was reached. Instead, the final subset of 20 review articles was selected purposively to ensure broad representation of the existing evidence and to reflect the diversity of perspectives across healthcare professions, geographical regions, methodological approaches, and major research themes. The characteristics of the selected studies are presented in Supplementary Table S1.

The selected review articles were systematically coded and examined according to a predefined analytical framework that included study population, data sources, methodological approach, principal findings, reported challenges, and acknowledged limitations. This structured synthesis complemented the quantitative bibliometric analyses by identifying recurrent themes, persistent knowledge gaps, and emerging research priorities that could not be fully captured through bibliometric indicators alone. To examine professional representation across the healthcare workforce, publications were classified according to the principal healthcare professional group investigated, based on the information reported in the title, abstract, author keywords, and, where necessary, the full bibliographic record available in the Web of Science Core Collection. The main categories included physicians, nurses, allied health professionals, administrative personnel, support staff, mixed healthcare populations, and other healthcare workforce groups. Publications including multiple professional groups without a clearly predominant study population were classified as mixed healthcare populations to avoid arbitrary assignment to a single occupational category and to ensure consistent application of the classification criteria across the dataset. To assess the spatial dimension of burnout research, we also analyzed the geographic distribution of publications and thematic foci across countries, identifying underrepresented regions and areas with infrastructure-driven disparities. Country-level publication outputs were derived from the author-affiliation metadata available in the Web of Science Core Collection records and analyzed using the Bibliometrix R package. Country productivity was calculated using the standard full-counting approach implemented in Bibliometrix, whereby each country represented in the author affiliations of a publication was credited with one publication. Consequently, internationally co-authored publications could contribute to more than one country in the country-level analyses [15,23].

The citation impact and authorship collaboration patterns were analyzed through descriptive statistics and inferential modeling. We used a multilevel negative binomial regression model to better understand the factors influencing research visibility [23,24,25,26]. The covariates included in the model were selected a priori because they represented standardized publication-level characteristics that were consistently available across the bibliographic records extracted from the Web of Science Core Collection. The model was intended as an exploratory analysis of associations between selected document characteristics and citation counts rather than as an exhaustive predictive model of citation accumulation. The multilevel regression analyses were estimated using an analytical dataset comprising 1062 publications, corresponding to the observations available for model estimation. Consequently, the effective analytical sample was smaller than the complete bibliometric dataset (*n* = 1232), which was retained for all descriptive bibliometric analyses. This model was chosen because of the count nature of citation data, the presence of overdispersion, and the nested structure of the data (e.g., articles nested within journals) [23,24,25,26].

The negative binomial distribution was preferred because citation counts are non-negative integers and are often overdispersed (variance > mean) [23,24]. Furthermore, since articles are nested within journals, a multilevel (random intercept) structure allows us to model between-journal variation [25,26].

Let *Y_ij_* denote the number of citations for article i in journal *j*;

*X_ij_* denote the vector of article-level predictors;

*u_j_* denote the random intercept for journal *j*, capturing unobserved heterogeneity.

We model the following:

$$Y_{ij}=NegBin\left(\mu_{ij},\theta \right)$$

$$\begin{array}{cc} \log\left(\mu_{ij}\right)=\beta_{0} & +\beta_{1}{Authors\_number}_{ij}+\beta_{2}{Title\_length}_{ij}+\beta_{3}{Keywords\_number}_{ij}\\ & +\beta_{4}{Keywords\_Plus\_number}_{ij}+\beta_{5}{Abstract\_length}_{ij}\\ & +\beta_{6}{References\_number}_{ij}+\beta_{7}{Pages\_number}_{ij}+u_{j} \end{array}$$

$$u_{j}\sim N(0,\sigma_{u}^{2})$$

where

*μij represents the expected citation count for article i in journal j;*
*θ represents the dispersion parameter;*
*βk represents fixed effects for article-level characteristics;*
*uj represents the journal-specific random effect.*

The log link function ensures the mean is positive. The journal-level random intercept $u_{j}$ captures shared characteristics or prestige effects within journals. This model evaluates how article features affect citation impact while considering journal-specific factors [22,23,24,25]. Given potential concerns about overdispersion, we also estimated a multilevel Poisson model for comparison [23,24,25,26]. In contrast, the Poisson model assumes that the outcome variable follows a Poisson distribution in which the mean equals the variance (equidispersion) [23]. Fixed effects captured the relationship between the predictors and the outcome, while random effects accounted for the data’s nested structure, allowing for variation in intercepts across clusters [25,26].

Model selection was based on the Akaike Information Criterion (AIC), Bayesian Information Criterion (BIC), log-likelihood, and deviance statistics. The multilevel Poisson and negative binomial models were formally compared using a likelihood-ratio test, with the Poisson model treated as the restricted specification and the negative binomial model as the specification allowing an additional dispersion parameter. The comparison yielded LR χ^2^(1) = 34,223 (*p* < 2.2 × 10^−16^), supporting the negative binomial model [23,24].

To assess model adequacy, we conducted residual diagnostics. The diagnostics included quantile-quantile (Q-Q) plots of residuals and residuals-versus-fitted-values plots. The Q-Q plot evaluated the agreement between the observed and expected residual distributions by showing deviations from the 45-degree line. The residuals-versus-fitted-values plots facilitated the detection of nonconstant variance or nonlinear relationships. Statistical tests for dispersion, outliers, and quantile deviations supported the graphical assessments [27].

Finally, we applied the DHARMa zero-inflation test to assess whether the fitted model adequately captured the proportion of zeros. This test compares the observed zeros to the expected number under the model’s simulations [27]. Although zero inflation was evaluated using the DHARMa simulation-based diagnostic procedure, the exact observed proportion of zero citations in the analytical dataset was not retained in the archived model output and therefore could not be reported retrospectively.

The PRISMA-style flow diagram refers exclusively to the purposive selection of the 20 review articles included in the structured critical literature synthesis and does not describe the retrieval of the bibliometric dataset used for the bibliometric analyses.

The multilevel regression analyses were performed using a complete-case approach; consequently, only publications with complete data for all variables included in the regression model were retained for model estimation (*n* = 1062), whereas descriptive bibliometric analyses were conducted using the complete bibliometric dataset (*n* = 1232).

## 3. Results

### 3.1. Results Related to the First Research Objective

**Figure 2:**
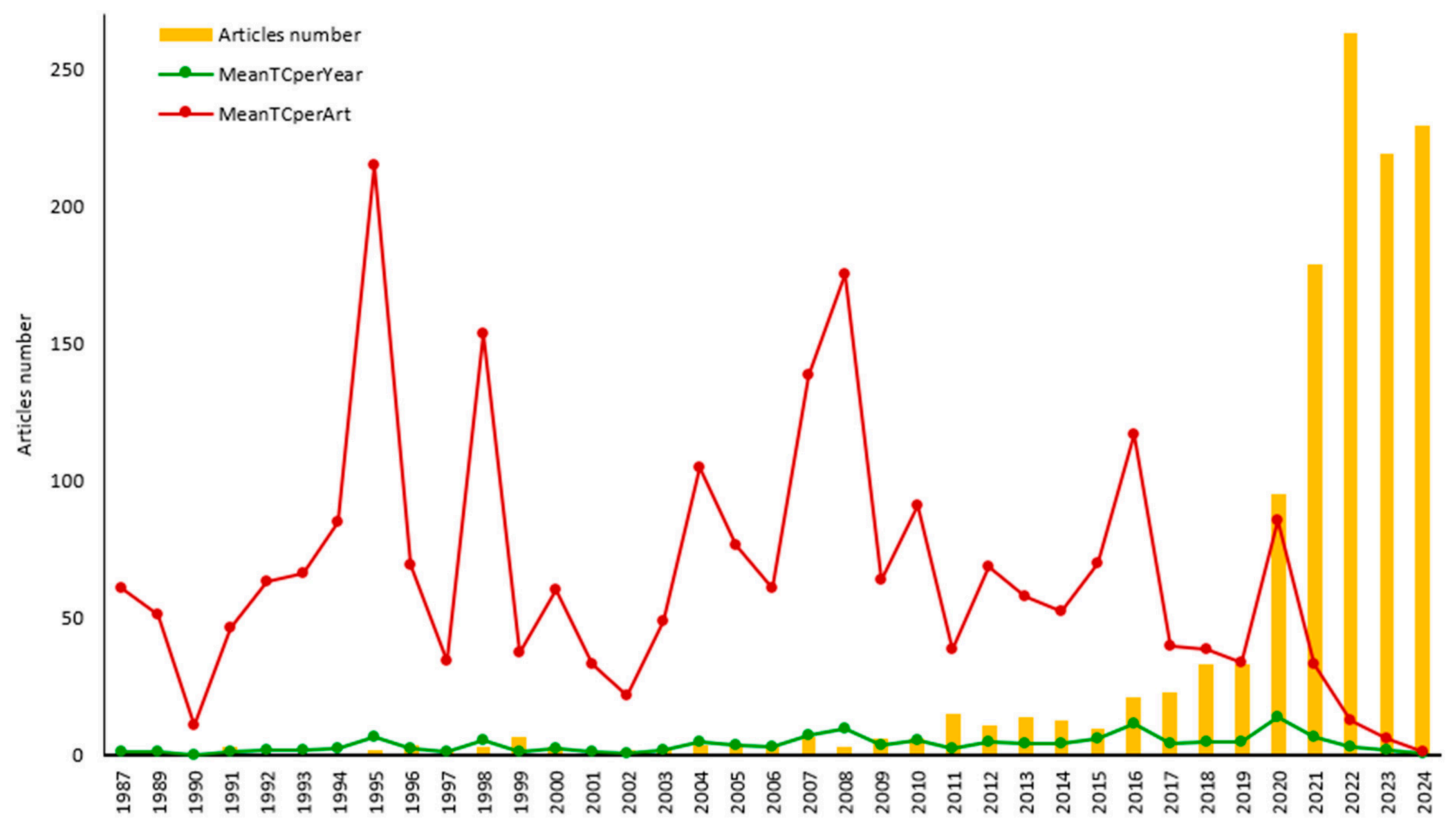
Annual scientific production and citation impact of burnout research among healthcare professionals (1987–2024). Yellow bars represent annual publication output, while the green and red lines indicate the mean citations per year and the mean citations per article, respectively. The average annual growth rate was 4.83%.

The analysis revealed a distinct three-phase evolution in scientific interest regarding occupational burnout among healthcare professionals. From 1987 to the early 2000s, research activity was minimal, marked by sporadic publications and limited visibility (Figure 2).

During this period, the high average citation rate per article reflects the influence of foundational studies that have shaped subsequent discourse. Between 2000 and 2015, there was a gradual yet consistent increase in publication volume, reflecting a growing academic engagement with varying citation impact. A significant inflection point occurred after 2020, coinciding with the COVID-19 crisis, when publications peaked dramatically in 2022. This sharp rise temporally coincided with the COVID-19 pandemic period and with increased scientific attention to healthcare workforce wellbeing. However, the recent decline in mean citations per article is likely due to a natural citation lag, as newer publications have not yet had time to accumulate citations. Overall, the findings demonstrate sustained scientific interest in burnout research and a marked expansion of publications during the period following 2020.

**Figure 3:**
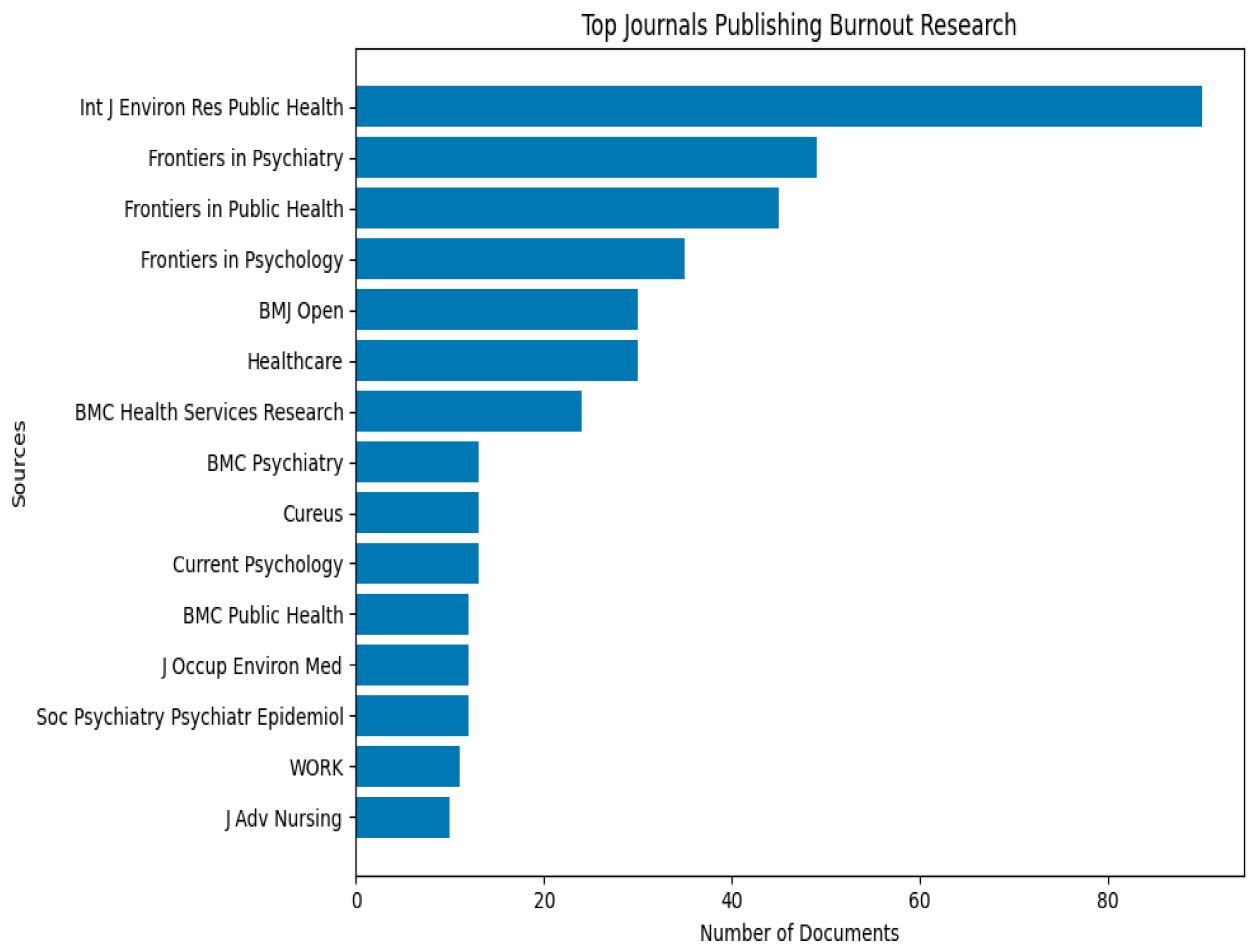
Top 15 journals publishing research on burnout among healthcare professionals (1987–2024). Bars represent the number of publications indexed in the Web of Science Core Collection for each journal during the study period.

The distribution of research output across journals reveals a multidisciplinary engagement with occupational burnout among healthcare professionals (Figure 3). The International Journal of Environmental Research and Public Health stands out as the leading journal, with 90 documents—nearly double the output of the next-ranked journal—underscoring its pivotal role in disseminating research on health-related workplace stress and mental well-being. This journal is followed by Frontiers in Psychiatry (49 documents), Frontiers in Public Health (45), and Frontiers in Psychology (35), which highlight the strong representation of psychological and public health perspectives. Journals such as BMJ Open, Healthcare, and BMC Health Services Research also feature prominently, indicating a balanced intersection of clinical, systemic, and organizational views. This distribution reflects the multidisciplinary nature of burnout research, encompassing public health, psychiatry, psychology, clinical medicine, and healthcare management.

Including specialized sources such as the Journal of Occupational and Environmental Medicine and Work: A Journal of Prevention, Assessment, and Rehabilitation underscores the relevance of occupational medicine and rehabilitation science. This diversity of publication venues reflects the multidisciplinary nature of burnout research among healthcare professionals.

**Figure 4:**
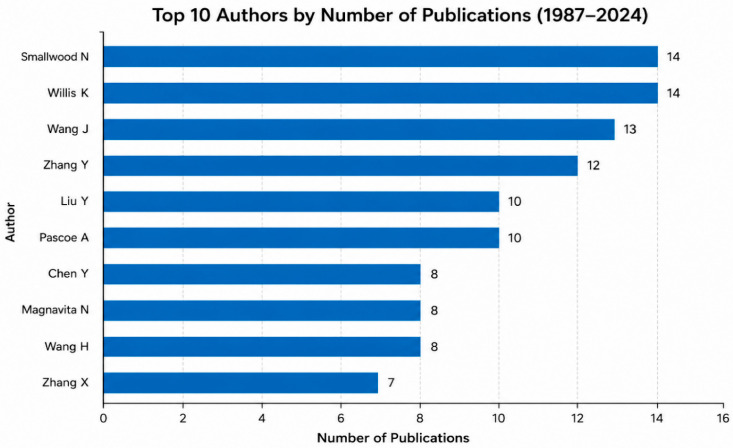
Top 10 most prolific authors in burnout research among healthcare professionals (1987–2024). Bars represent the number of publications indexed in the Web of Science Core Collection.

The analysis of author productivity highlights a group of researchers who have made significant contributions to the literature on occupational burnout in healthcare settings (Figure 4). Smallwood N and Willis K lead with 14 publications each, indicating sustained research engagement in the field of occupational burnout among healthcare professionals. Wang J follows closely with 13 publications, followed by Zhang Y, with 12, reflecting a substantial contribution from authors affiliated with Chinese research institutions. Other key contributors, such as *Liu Y* and *Pascoe A* (10 publications each) and *Chen Y*, *Magnavita N*, and *Wang H* (each with 8), further demonstrate a diverse pool of researchers spanning psychology, public health, and occupational medicine. The recurring presence of common surnames (e.g., Wang and Zhang) may also reflect multiple prolific researchers or collaborative research groups within these academic networks. These findings indicate sustained research activity by a relatively small group of highly productive authors who have contributed substantially to the development of the field.

**Figure 5:**
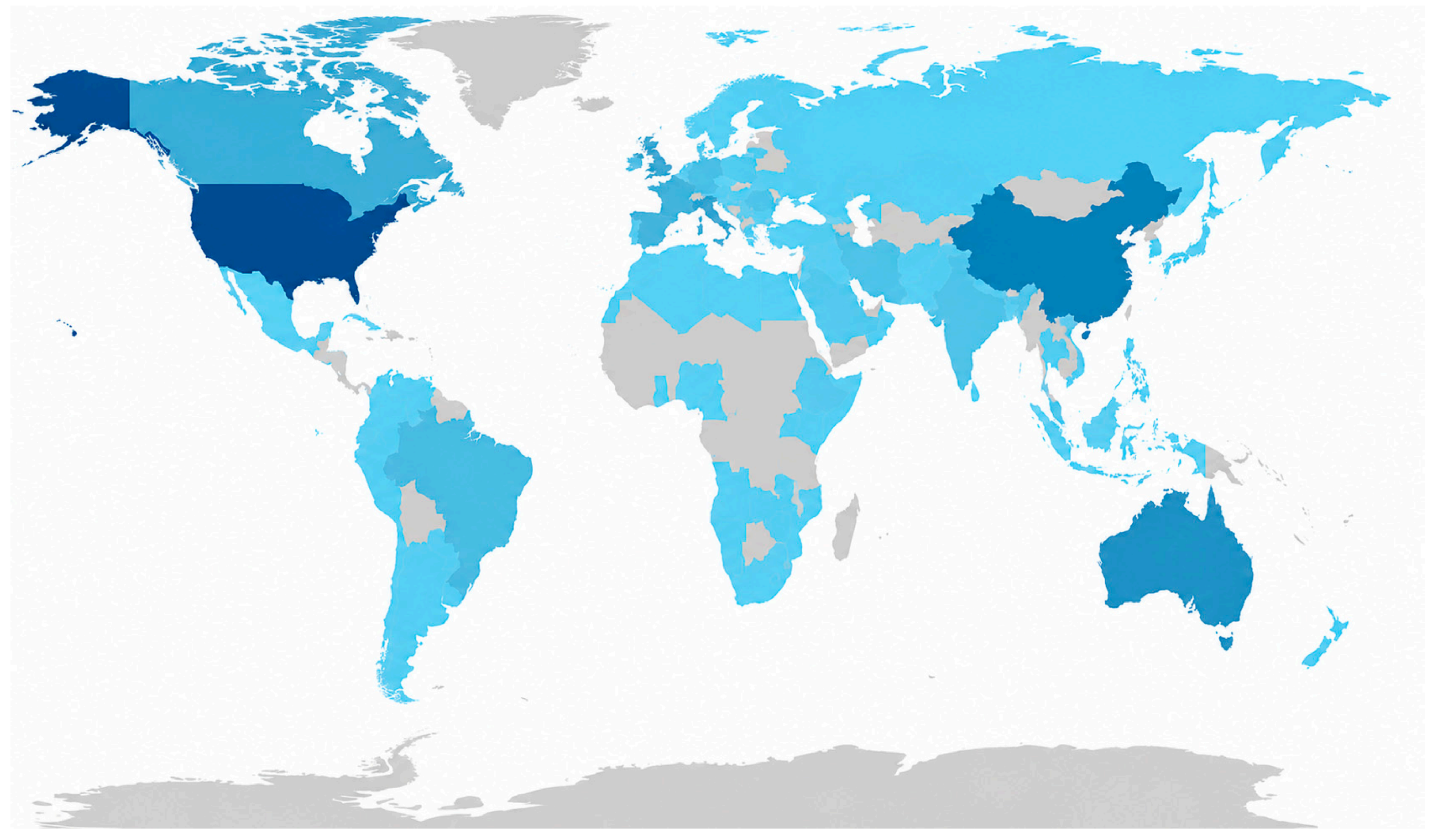
Colour intensity reflects the number of publications identified for each country. Darker shades indicate higher scientific production (greater number of publications indexed in Web of Science), whereas lighter shades indicate lower publication output. Grey areas represent countries with no publications identified in the dataset.

The geographic distribution of publications reveals substantial disparities in global research activity on occupational burnout among healthcare professionals (Figure 5).

The United States and China stand out as the most prolific contributors, reflecting their extensive research infrastructures and acute awareness of challenges in the healthcare workforce. Other notable contributors include Australia, Canada, the United Kingdom, and Germany, indicating strong academic involvement from high-income countries with advanced healthcare systems. The observed distribution of publications indexed in the Web of Science Core Collection indicates that the available literature is concentrated in countries with well-established research infrastructures and high publication capacity. This pattern should be interpreted considering the database coverage and English-language restrictions applied in the present study.

Regions such as Latin America (e.g., Brazil and Argentina), South and Southeast Asia (e.g., India, South Korea), and Sub-Saharan Africa demonstrate moderate research activity, suggesting a growing awareness and capacity for investigation in emerging economies. However, significant gaps remain in the Web of Science-indexed literature across Central Africa, parts of the Middle East, and some Eastern European countries. This limited visibility may reflect disparities in research capacity, publication opportunities, or database coverage rather than an absence of burnout-related research activity. This geographic pattern highlights the global relevance of healthcare burnout while revealing disparities in research output. These findings highlight the importance of broader international collaboration and improved representation of underrepresented regions in future research on healthcare workforce burnout.

### 3.2. Results Related to the Second Research Objective

**Figure 6:**
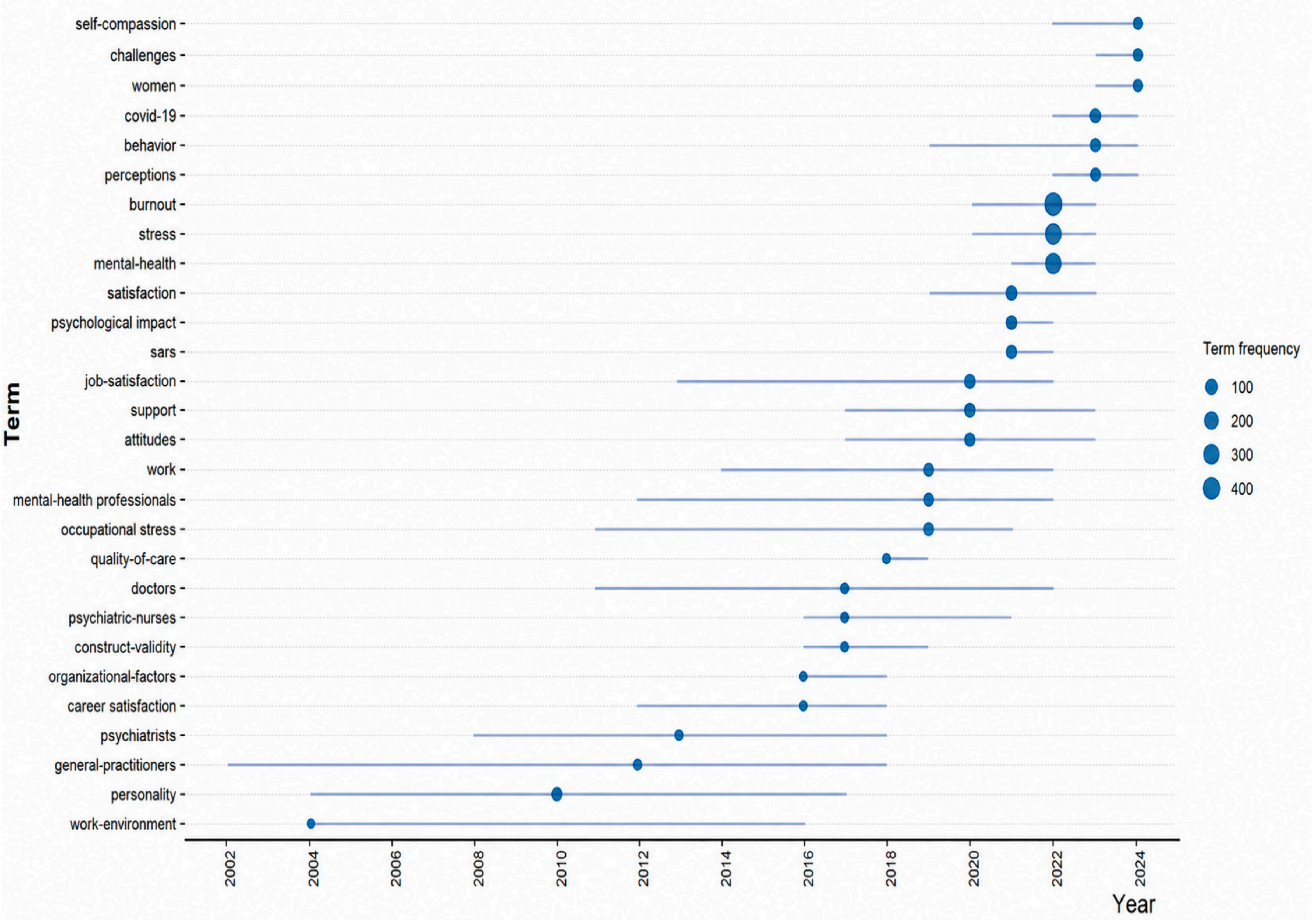
Temporal evolution of major research topics in burnout research among healthcare professionals (1987–2024). The thematic evolution analysis was generated using the Bibliometrix package in R from publications retrieved from the Web of Science Core Collection published between 1987 and 2024. The analysis illustrates the evolution of the bibliographic metadata selected for thematic analysis across consecutive time periods. Node size reflects the relative prominence of each term within the corresponding period. Because this visualization represents thematic evolution rather than a network structure, edge definitions and network normalization are not applicable.

Figure 6 illustrates the evolution of research priorities and emerging areas of interest across different phases of burnout scholarship.

**Figure 7:**
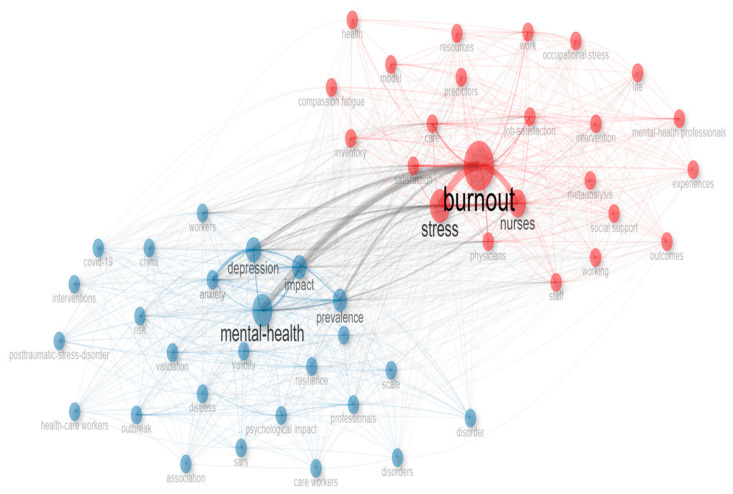
Keyword co-occurrence network illustrating the conceptual structure of burnout research among healthcare professionals. The network was generated using VOSviewer (version 1.6.20) from publications retrieved from the Web of Science Core Collection (1987–2024). The analysis was based on the bibliographic metadata field selected according to the predefined analytical procedure described in the Section 2. Nodes represent individual keywords, node size reflects keyword occurrence frequency, links indicate keyword co-occurrence relationships, and link thickness reflects the relative strength of co-occurrence between terms. The network visualization was generated using the association-strength normalization implemented in VOSviewer.

Early research (before 2015) focused on foundational constructs such as the work environment, personality traits, psychiatrists, and general practitioners, highlighting an initial emphasis on individual characteristics and specific professional subgroups. From 2015 to 2020, the field broadened to include organizational factors, construct validity, and career satisfaction, suggesting an increasing research interest in systemic and occupational determinants of burnout. Terms such as occupational stress, mental health professionals, and quality of care became more prominent during this period, suggesting a broader thematic emphasis on organizational and healthcare system contexts alongside individual-level perspectives. After 2020, coinciding with the COVID-19 pandemic, acute stressors and psychosocial responses became increasingly prominent research topics. The most prominent contemporary terms, including COVID-19, mental health, satisfaction, psychological impact, support, and self-compassion, suggest a thematic emphasis on emotional resilience, workplace challenges, and coping mechanisms during the pandemic period. Notably, gender-related themes emerged as an increasingly visible area of research, highlighting differential burnout experiences and occupational challenges among healthcare professionals during public health crises. The co-occurrence analysis reveals the conceptual architecture of burnout research and the relationships among its dominant thematic domains (Figure 7).

Two distinct clusters emerge, indicating major thematic domains. The red cluster, centered around burnout, includes closely related terms such as stress, nurses, occupational stress, intervention, job satisfaction, and mental health professionals. This grouping focuses on work-related stressors, professional roles, and organizational factors affecting emotional exhaustion and workforce performance. The blue cluster, anchored by mental health and depression, incorporates terms like anxiety, prevalence, psychological impact, healthcare workers, and COVID-19. This cluster emphasizes psychological outcomes, diagnostic considerations, and public health concerns, particularly during crises like the pandemic. The strong connections between the clusters (e.g., burnout, mental health, and depression) indicate the interdisciplinary nature of burnout research, where occupational health, psychology, psychiatry, and public health intersect. The prominence of terms such as resilience, support, and intervention also suggests increasing research interest in protective factors and intervention approaches.

**Figure 8:**
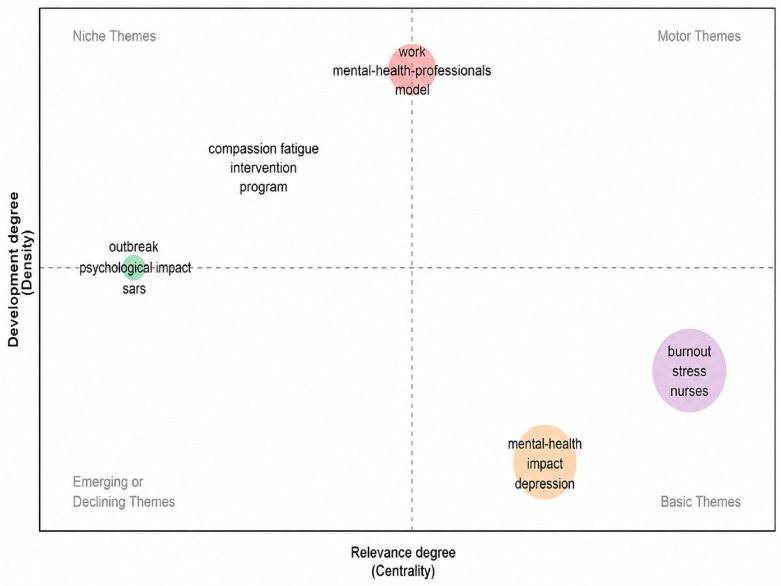
Thematic map of burnout research among healthcare professionals. The thematic map was generated using the Bibliometrix package in R from publications retrieved from the Web of Science Core Collection (1987–2024). Themes were identified using the thematic mapping procedure implemented in Bibliometrix according to the predefined analytical settings described in Section 2. Each bubble represents a thematic cluster, and bubble size reflects the relative frequency of the terms included in that cluster. The horizontal axis (centrality) represents the degree of interaction between themes, whereas the vertical axis (density) reflects the internal development and cohesion of each thematic cluster. Because the thematic map summarizes thematic clusters rather than network links, edge definitions are not applicable.

The thematic map (Figure 8) categorizes research topics on occupational burnout among healthcare professionals along two dimensions: centrality (relevance to the field) and density (development/maturity of the theme). Four thematic quadrants can be identified:

▪ Motor themes: The cluster including work, mental health professionals, and models occupies the top-right quadrant, indicating a well-developed and influential thematic area. These concepts are structurally essential and drive the research agenda, often as theoretical or methodological anchors.
▪ Basic themes: The largest and most prominent cluster (burnout, stress, nurses) resides here. These terms are foundational and widely discussed, signifying core concerns in the literature. Despite their central role, the relatively low density indicates that these themes remain broadly developed and may continue to evolve conceptually.
▪ Niche themes: Topics such as compassion fatigue, intervention, and programs are well-developed but marginal regarding broader field influence. These themes represent specialized research areas or targeted applications, often within specific populations or intervention strategies.
▪ Emerging or declining themes: These themes were particularly relevant during the early stages of the COVID-19 pandemic or may represent emerging research areas that have not yet become fully integrated into the mainstream burnout literature.

The thematic structure suggests that burnout research has evolved around a stable core of occupational stress and nursing-related studies while simultaneously expanding toward mental health, resilience, and intervention-oriented research.

### 3.3. Results Related to the Third Research Objective

The structured synthesis identified several recurrent gaps in the evidence base, including the predominance of studies involving physicians and nurses, limited representation of non-clinical healthcare workers and low- and middle-income settings, methodological heterogeneity in burnout measurement, the widespread use of cross-sectional designs, and the limited evaluation of organizational or system-level interventions. Emerging areas requiring further investigation included occupational differences, gender-related experiences, physiological correlates of burnout, and the longer-term consequences of pandemic-related occupational strain [4,5,6,8,9,11,14].

### 3.4. Results Related to the Fourth Research Objective

The distribution of the study variables was evaluated before fitting the multilevel negative binomial regression model. As shown in Supplementary Table S2, the summary statistics and graphs indicate that many variables are not normally distributed, as they have a strong positive skew and high kurtosis. Citations_number, Pages_number, Abstract_length, Authors_number, and References_number have uneven distributions with extreme values and long right tails. These findings suggest that while most articles fall within typical ranges, a few stand out with exceptionally high values. In contrast, Title_length and Keywords_number display near-symmetric distributions with minimal skewness, while Keywords_plus_number shows a slightly negative skew and a flat distribution, indicating a relatively even spread around the mean.

The two multilevel models presented in Table 3 estimate expected citation counts while accounting for journal-level clustering. The substantially better fit of the negative binomial model relative to the Poisson model, together with the estimated dispersion parameter (0.751) and the likelihood-ratio test (LR χ^2^(1) = 34,223, *p* < 2.2 × 10^−16^), indicated that the Poisson equidispersion assumption was not compatible with the observed citation data.

The results in Table 3 compare two multilevel count models: a Poisson model and a negative binomial model. The negative binomial model provided a significantly better fit to the data than the Poisson model, as indicated by the model comparison test, confirming the presence of substantial overdispersion in the outcome variable. In the negative binomial model, the variables Title_length, Keywords_number, and Keywords_plus_number were statistically significant. Specifically, longer titles and a higher number of author-provided keywords were associated with lower expected citation counts, whereas a greater number of Keywords Plus terms was positively associated with citation impact. The remaining predictors (author count, abstract length, page number, and reference count) were not statistically significant.

In contrast, although the Poisson model indicated that all predictors were statistically significant, its substantially poorer fit (higher AIC and BIC values) suggests that its underlying assumptions were less compatible with the observed data.

**Figure 9:**
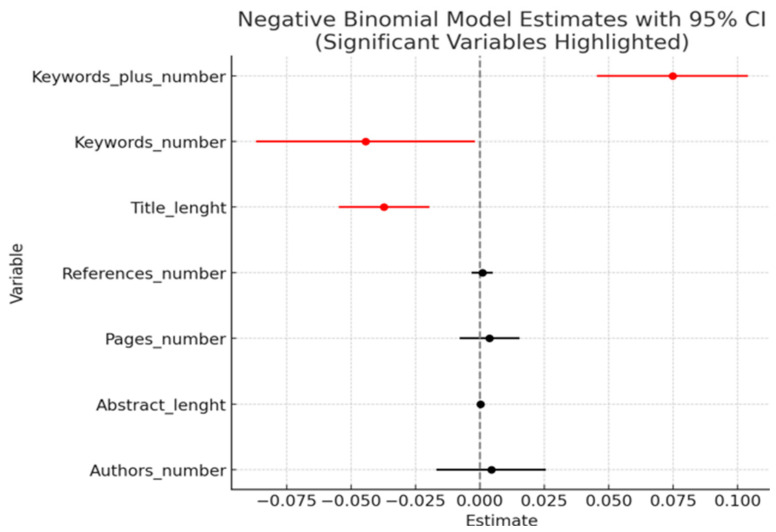
Forest plot showing the regression coefficients (β) and their 95% confidence intervals for the predictors included in the multilevel negative binomial model. Variables with statistically significant effects (*p* < 0.05) are highlighted in red. Specifically, longer titles and a greater number of author-provided keywords were significantly associated with lower expected citation counts. In contrast, a greater number of Keywords Plus terms was positively associated with citation impact. The remaining predictors (number of authors, abstract length, page count, and reference count) were not statistically significant, as their 95% confidence intervals crossed zero. The residual diagnostics for the multilevel negative binomial model (Figure 10 and Figure 11) were evaluated using graphical and simulation-based diagnostic procedures implemented in the DHARMa package to assess potential departures from the underlying model assumptions.

Overall, the negative binomial model was selected as the final model because it provided the best fit to the data. These findings indicate that specific document characteristics, particularly title length, author-provided keywords, and Keywords Plus terms, were significantly associated with citation counts. Figure 9 presents the regression estimates and 95% confidence intervals for each predictor included in the multilevel negative binomial model. To facilitate the interpretation of the regression coefficients, the statistically significant predictors may also be expressed in terms of their practical effect on the expected citation counts. Specifically, each additional word included in the article title was associated with an approximate 3.7% decrease in the expected number of citations. Likewise, each additional author-provided keyword was associated with an approximate 4.3% decrease in expected citation counts. In contrast, each additional Keywords Plus term was associated with an approximate 7.7% increase in the expected number of citations. These percentage changes were obtained by exponentiating the regression coefficients from the negative binomial model and are presented to facilitate the practical interpretation of the observed associations [23,24,25,26].

The Q–Q plot indicated departures from the expected residual distribution, as reflected by the statistically significant Kolmogorov–Smirnov uniformity test. These findings suggest that the residual distribution did not perfectly conform to the theoretical expectation. Accordingly, the regression findings should be interpreted with appropriate caution.

The residuals-versus-predicted plot indicated departures in the upper quantiles at higher predicted values. Although dispersion, outlier, zero-inflation, and quantile diagnostics were also evaluated using the DHARMa package, the graphical assessment suggests localized deviations across the range of predicted values. Consequently, the regression results should be interpreted with appropriate caution.

**Figure 10:**
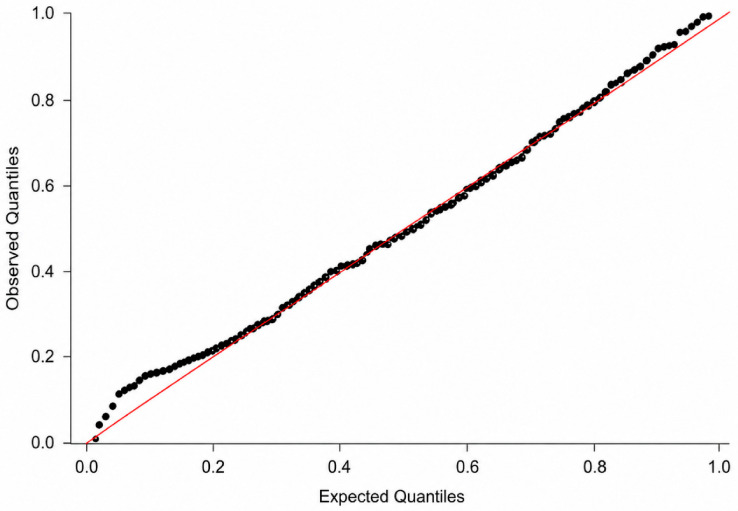
Q–Q plot of simulated residuals from the multilevel negative binomial regression model. The figure illustrates the agreement between the observed and expected residual quantiles and allows visual assessment of departures from the theoretical residual distribution. Points represent simulated DHARMa residuals; colored lines represent the estimated conditional quantiles, and dashed horizontal lines indicate the expected quantiles under the fitted model.

**Figure 11:**
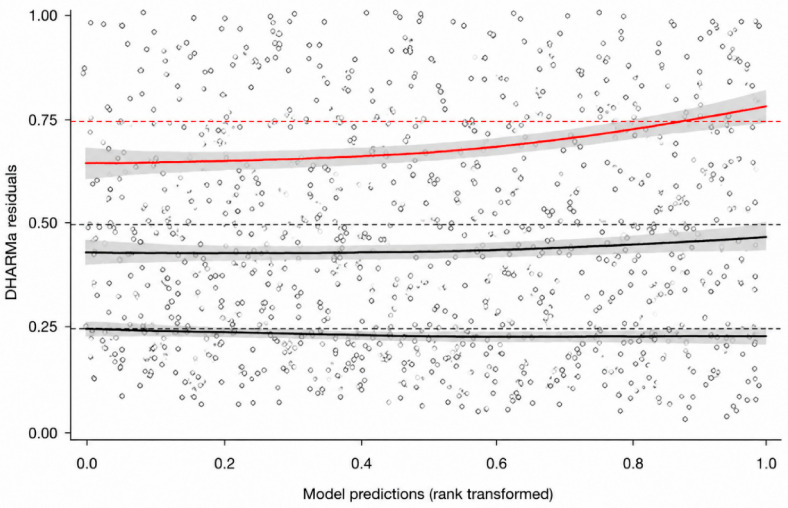
DHARMa residuals plotted against model predictions for the multilevel negative binomial regression model. The figure displays simulated residuals, quantile regression lines, and confidence bands used to assess model fit and potential deviations from model assumptions. Open circles represent the simulated DHARMa residuals; colored solid lines indicate the estimated quantile regression lines, dashed horizontal lines represent the expected quantiles under the fitted model, and the gray shaded areas denote the corresponding confidence bands.

## 4. Critical Synthesis of Global Burnout Research

### 4.1. From Individual Distress to Systemic Vulnerability

One of the most important developments observed across four decades of burnout research is the gradual transition from an individual-centered perspective toward a systems-oriented understanding of occupational burnout [1,4]. Early studies primarily conceptualized burnout as a psychological response to prolonged occupational stress, emphasizing personal resilience, coping strategies, and individual vulnerability [1]. Over time, however, accumulating evidence has increasingly demonstrated that burnout is strongly influenced by organizational and structural factors [1,4].

The thematic evolution identified in the present study reflects this transition. Concepts such as workload, staffing adequacy, organizational support, leadership quality, workplace culture, and institutional resources have become increasingly prominent in the literature [1,4,11,12,13]. These findings suggest that burnout should not be understood solely as a failure of individual adaptation but rather as an indicator of broader organizational dysfunction [1,4,11,12,13]. Within this context, burnout emerges as a marker of workforce vulnerability and potentially, institutional fragility [1,4].

### 4.2. The Dominance of Clinical Professions in Burnout Research

A second major observation concerns the concentration of research attention on physicians and nurses. These professional groups account for the majority of publications identified across the study period and continue to dominate contemporary burnout research [10,11,19,20].

While this focus is understandable given their central role in patient care, broader representation of allied health professionals, administrative personnel, support staff, and other underrepresented occupational groups is needed to improve understanding of burnout across the healthcare workforce [4,5,6].

This imbalance limits the current understanding of burnout across the broader healthcare workforce [4,5,6].

### 4.3. Geographic Inequalities and the Global Evidence Gap

The geographic distribution of publications reveals substantial inequalities in the global production of knowledge regarding burnout among healthcare professionals. Research activity remains concentrated in North America, Western Europe, Australia, and selected Asian countries, while many low- and middle-income regions contribute relatively little to the indexed literature [5,6,8,21].

Importantly, limited publication output should not be interpreted as evidence of a lower burden of burnout. Rather, it may reflect disparities in research infrastructure, funding availability, academic resources, publication opportunities, and database representation. Consequently, the current evidence base may disproportionately reflect experiences from highly resourced healthcare systems while underrepresenting settings where workforce shortages, resource constraints, and occupational pressures may be even more severe [5,21].

These findings emphasize the importance of improving the geographical representativeness of future burnout research, particularly through greater inclusion of low- and middle-income settings [4,21].

### 4.4. Persistent Methodological Challenges

Despite considerable growth in scientific output, several methodological limitations continue to characterize the field. One of the most persistent challenges concerns the heterogeneity of burnout measurement. The widespread use of different burnout assessment instruments and operational definitions has generated difficulties in comparing prevalence estimates and synthesizing findings across studies [10,14].

Additional limitations include the predominance of cross-sectional designs, reliance on self-reported measures, limited longitudinal follow-up, and variability in burnout definitions and reporting standards. These methodological inconsistencies contribute to substantial variation in reported prevalence rates and complicate efforts to establish robust evidence regarding causal pathways and intervention effectiveness [10,11,12,13,14].

Greater methodological consistency remains essential to improve the comparability and reproducibility of future burnout research [10,14].

### 4.5. From Problem Identification to Intervention Science

One of the most important findings of the present study is the imbalance between studies describing burnout and those evaluating potential solutions. The literature has generated extensive evidence regarding prevalence, risk factors, psychological consequences, and occupational correlates of burnout [5,6,7,8,9,10,11,12,13,14]. In contrast, relatively few studies have examined the effectiveness of organizational interventions, workforce policies, leadership strategies, staffing models, or system-level reforms [12,13]. Future research should increasingly focus on intervention science, implementation research, and organizational transformation strategies capable of improving workforce wellbeing at scale [4,12,13].

### 4.6. Burnout and the Future Sustainability of Healthcare Systems

The accumulated evidence reviewed in this study indicates that the scientific literature increasingly conceptualizes burnout as a broader organizational and health-system challenge extending beyond an individual occupational health issue [1,3,4,11,14,28].

As healthcare systems worldwide continue to face demographic ageing, workforce shortages, increasing service demands, and financial pressures, understanding and addressing burnout will become increasingly important [1,4]. Future burnout research should increasingly focus not only on documenting occupational distress but also on identifying effective strategies to strengthen workforce resilience, support organizational adaptation, and enhance the long-term sustainability of healthcare systems [4,12,13].

## 5. Discussion

The present study provides a comprehensive overview of the evolution of burnout research among healthcare professionals over nearly four decades. By integrating bibliometric mapping, citation modelling, and critical synthesis of the literature, the study offers insights not only into what is currently known about burnout but also into how scientific attention has evolved and where important gaps persist.

One of the most striking findings is the remarkable expansion of burnout research after 2020. Although concerns regarding occupational stress and professional wellbeing have existed for decades, the marked increase in publications after 2020 temporally coincided with the COVID-19 pandemic and with growing scientific attention to healthcare workforce wellbeing [6,7,8]. The rapid increase in publications reflects growing recognition that healthcare workforce wellbeing is not a peripheral issue but a fundamental component of health system performance and resilience [1,3,6].

The thematic evolution observed throughout the study period suggests a broader conceptual evolution within the field. Early investigations focused primarily on individual psychological responses, whereas more recent publications increasingly emphasize organizational, managerial, and systemic determinants [1,4,11,12]. This pattern is consistent with the maturation of the field; however, the present bibliometric analyses were not designed to quantify the magnitude or statistical significance of this thematic evolution [1,4]. Burnout is no longer viewed exclusively as a personal inability to cope with workplace stress but rather as a complex phenomenon shaped by staffing levels, leadership quality, organizational culture, administrative burden, and resource availability [1,4,11,12].

Recent evidence also suggests that digital work environments represent an emerging organizational determinant of burnout. Systematic reviews examining electronic health record (EHR) use have reported that increased documentation requirements, cognitive workload, workflow interruptions, and reduced time available for direct patient care may contribute to emotional exhaustion and professional dissatisfaction among clinicians. These findings reinforce the view that burnout should also be considered within the context of healthcare digitalization, where technological systems may function both as potential sources of occupational strain and as targets for organizational improvement [29,30].

Another important finding of this study concerns the uneven geographic distribution of publications within the indexed evidence base. The publications indexed in the Web of Science Core Collection and retrieved in the present study were concentrated in high-income countries, particularly the United States, China, the United Kingdom, Canada, and Australia [19,20,21]. However, this pattern should be interpreted cautiously because it may reflect not only differences in research productivity, but also the database coverage and English-language restrictions applied in the present study. The lower representation of many low- and middle-income regions therefore limits the global representativeness of the indexed evidence base [5,15,21]. Healthcare workers operating in resource-constrained environments may face challenges that differ significantly from those reported in highly resourced systems [5,6]. Future research should therefore include broader database coverage, multilingual search strategies, and greater geographical diversity to improve the representativeness of the evidence base [4,15,21].

Although burnout research has expanded substantially, comparatively fewer studies have evaluated organizational and system-level interventions capable of producing sustainable improvements. This imbalance highlights an important priority for future research [4,12,13].

The findings of the citation analysis provide an additional perspective on knowledge dissemination within the field and address one of the study objectives by identifying publication characteristics associated with citation impact and scientific visibility. The observed associations between publication characteristics and citation counts highlight the potential importance of scientific communication and article visibility. However, these findings should not be interpreted as evidence of causal relationships, because the multilevel regression model was observational and evaluated statistical associations rather than causal effects. At the same time, citation counts should not be interpreted as direct indicators of scientific quality. Moreover, the positive association observed for Keywords Plus should be interpreted cautiously because these terms are generated algorithmically from cited-reference information within the Web of Science database rather than being directly selected by authors. Consequently, the observed association may partly reflect indexing characteristics and information retrieval processes rather than an independent relationship with citation impact [15,17]. Citation dynamics are influenced by multiple factors, including journal visibility, indexing practices, publication age, disciplinary trends, and evolving research priorities. Although the observed effect sizes were modest at the individual variable level, they suggest that relatively small differences in publication characteristics may be associated with differences in the scientific visibility of articles [15,16].

In addition, although the multilevel negative binomial model identified several publication characteristics associated with citation impact, publication age was not explicitly incorporated as an exposure variable. Consequently, older publications had a longer opportunity to accumulate citations than more recent publications. Therefore, the reported associations should be interpreted as exploratory rather than causal estimates of citation determinants. Future bibliometric studies should incorporate publication year or citation-exposure adjustments to better account for temporal differences in citation accumulation [23,24,25,26]. The journal-level random intercept accounted for between-journal heterogeneity but did not fully control for all potential article-level determinants of citation accumulation. Additional factors such as document type, journal visibility, research field, open-access status, international collaboration, and article topic may also have contributed to the observed citation patterns. Because these variables were not incorporated simultaneously into the exploratory model, residual confounding cannot be excluded [15,23,24,25,26].

Taken together, the bibliometric findings indicate that contemporary burnout research increasingly addresses workforce sustainability, patient safety, organizational performance, and health-system resilience as major themes within the scientific literature [1,3,4]. These findings describe the evolution of research priorities rather than demonstrating the direct consequences of burnout itself.

Several limitations should be acknowledged. The analysis was restricted to publications indexed in the Web of Science Core Collection and to English-language literature. Consequently, relevant studies indexed in other bibliographic databases or published in other languages may not have been captured. These methodological choices may have introduced database, language, and regional coverage bias. Therefore, the geographic distribution reported in this study reflects the indexed literature retrieved under the predefined methodological criteria rather than the entirety of global burnout research. The observed concentration of publications originating from high-income countries should therefore be interpreted with caution, as it may partially reflect database coverage and indexing policies in addition to genuine differences in research productivity [15]. Furthermore, publication age was not explicitly incorporated as an exposure variable in the multilevel citation model; therefore, the observed associations with citation impact should be interpreted with appropriate caution [23,24,25,26]. Moreover, the covariate specification did not include all potential determinants of citation accumulation, such as document type, journal visibility, research field, open-access status, international collaboration, and article topic. Formal sensitivity analyses using alternative covariate specifications were beyond the scope of the present exploratory bibliometric study and should be considered in future investigations. Therefore, the robustness of the reported associations to residual confounding should be interpreted with appropriate caution. Future bibliometric studies should evaluate these associations using broader covariate specifications and formal sensitivity analyses [15,23,24,25,26]. Furthermore, the multilevel regression analyses were estimated using an analytical dataset comprising 1062 publications. Consequently, the inferential analyses were based on a smaller analytical sample than the complete bibliometric dataset (*n* = 1232), and this difference should be considered when interpreting the regression findings. In addition, the exact numerical outputs of the DHARMa diagnostic tests, including the uniformity, dispersion, outlier, zero-inflation, and quantile-deviation statistics, were not available for retrospective reporting. Consequently, the magnitude of the diagnostic deviations could not be quantified, and conclusions regarding model adequacy should be interpreted with appropriate caution [15,23,24,25,26]. Although residual diagnostics were performed using graphical and simulation-based procedures, the observed departures from the expected residual distribution indicate that the regression findings should be interpreted with appropriate caution. Future studies should further evaluate model adequacy using complementary diagnostic approaches and alternative model specifications [23,24,25,26,27].

In addition, the Web of Science Core Collection provides a standardized and high-quality bibliographic dataset but does not include all relevant publications indexed in other international or regional databases. Consequently, the use of a single bibliographic database may have limited the inclusion of relevant publications indexed exclusively in Scopus, PubMed, Embase, CINAHL, PsycINFO, or regional databases, potentially influencing the representation of certain countries, institutions, and research themes [15,16,17,18]. Furthermore, different combinations of search terms may retrieve partially different bibliographic datasets. Although alternative search expressions (e.g., “job burnout”, “professional burnout”, “occupational burnout”, and “emotional exhaustion”) may retrieve a partially different set of records, the final search strategy was selected to provide a consistent and reproducible bibliographic dataset aligned with the objectives of the present bibliometric study. Similarly, although broad umbrella descriptors were selected to maximize retrieval across the healthcare workforce, some studies indexed exclusively under profession-specific terminology may not have been identified.

The exclusion of the search terms “students” and “educators” may also have resulted in the omission of publications involving resident or trainee physicians, whose experiences may differ from those of the actively employed healthcare workforce. Furthermore, although a retracted publication identified in the dataset was retained to preserve the transparency and completeness of the bibliometric analysis, its inclusion may have had a limited influence on the citation-based analyses. Nevertheless, the large dataset, the extended study period, and the integration of bibliometric mapping, critical literature synthesis, and multilevel citation modelling provide a comprehensive overview of burnout research among healthcare professionals. Overall, this study suggests that burnout research has entered a new phase characterized by growing recognition of structural determinants, increasing attention to workforce sustainability, and a gradual shift toward organizational and system-level solutions [1,4,12,13]. The challenge for future research is not merely to document burnout more extensively, but to generate actionable evidence capable of supporting meaningful improvements in healthcare workforce wellbeing and health system resilience [4,12,13]. Building on these findings, several priorities emerge that may guide future research and further strengthen the evidence base on burnout among healthcare professionals.

### 5.1. Future Research Directions

Based on the findings of the present study, several priorities emerge that may guide future research in this field.

The rapid expansion of burnout research among healthcare professionals has generated a substantial body of evidence. However, important conceptual, methodological, geographic, and practical gaps continue to limit the development of a more coherent and actionable knowledge base. Addressing these limitations should remain a priority for future research and policy development [4,10,21].

#### 5.1.1. Standardization of Burnout Measurement

One of the most persistent challenges concerns the lack of standardized burnout measurement approaches. Future studies should prioritize methodological harmonization, transparent reporting standards, and greater comparability across burnout assessment instruments to strengthen evidence synthesis and international comparisons [10,14].

#### 5.1.2. Need for Longitudinal Research

The predominance of cross-sectional studies limits understanding of the temporal evolution and causal mechanisms of burnout. Future longitudinal investigations are needed to clarify burnout trajectories and their long-term consequences for workforce retention, professional performance, and health outcomes [11,14].

#### 5.1.3. Expanding Research Beyond Physicians and Nurses

Future research should adopt a broader workforce perspective by increasing the representation of allied health professionals, administrative personnel, support staff, healthcare managers, technical personnel, and other underrepresented occupational groups [4,6,10,11,14].

#### 5.1.4. Addressing Global Research Inequalities

Future international collaborations should strengthen the representation of low- and middle-income countries and support the generation of context-specific evidence capable of informing locally relevant workforce policies and organizational interventions [4,5,21].

#### 5.1.5. Strengthening Organizational and System-Level Intervention Research

Future research should place greater emphasis on evaluating organizational interventions, staffing models, leadership strategies, workload management, health policy reforms, and implementation research to strengthen the evidence supporting sustainable organizational change [4,12,13].

#### 5.1.6. Gender, Diversity, and Equity Perspectives

Emerging evidence indicates that burnout experiences may differ according to gender, professional role, career stage, and social context. However, many studies continue to report aggregated findings without examining these potentially important differences [6,7,8].

Future research should incorporate sex- and gender-sensitive analyses and consider broader equity dimensions, including ethnicity, socioeconomic status, and professional hierarchy [4,6].

#### 5.1.7. Digital Transformation and Emerging Sources of Occupational Stress

Healthcare systems are undergoing rapid digital transformation through electronic health records, telemedicine platforms, artificial intelligence tools, and data-intensive administrative systems. While these innovations have the potential to improve efficiency, they may also introduce new forms of occupational stress, administrative burden, and cognitive overload.

Future studies should explore the relationship between digitalization, technology-related workload, professional autonomy, and burnout outcomes across different healthcare settings [29,30].

#### 5.1.8. Burnout as a Workforce Sustainability Challenge

One of the most important priorities for future research involves integrating burnout research into broader discussions of workforce sustainability. Future bibliometric and empirical studies should further examine the relationships between burnout, workforce sustainability, organizational performance, and health-system resilience across diverse healthcare settings [1,3,4,14].

This broader perspective may help inform strategies that simultaneously improve employee wellbeing, strengthen organizational effectiveness, and support the long-term sustainability of healthcare systems [4,12,13].

## 6. Conclusions

This study provides a comprehensive assessment of the global development of burnout research among healthcare professionals between 1987 and 2024. By integrating bibliometric mapping, citation analysis, and critical synthesis of the literature, the study offers a multidimensional perspective on how scientific understanding of burnout has developed over time.

The findings demonstrate that burnout research has expanded substantially, particularly during and after the COVID-19 pandemic, while increasingly emphasizing organizational and health-system determinants alongside continued attention to individual-level perspectives. Nevertheless, important gaps persist regarding geographic representation, workforce diversity, methodological consistency, and the evaluation of organizational interventions. Addressing these limitations will be essential for improving the quality, comparability, and practical relevance of future burnout research.

Overall, the present bibliometric study demonstrates that scientific interest in burnout among healthcare professionals has expanded substantially and increasingly encompasses organizational and health-system perspectives. The findings identify important gaps in the existing literature and provide a framework for guiding future empirical research rather than establishing the prevalence, consequences, or effectiveness of interventions related to burnout.

## Figures and Tables

**Table 1:** Comparison of representative bibliometric studies on burnout among healthcare professionals and the present study.

Study	Population	Main Analytical Focus	Principal Limitation
Sweileh, 2020 [21]	Healthcare providers	Publication trends, research themes, citation analysis	Scopus database only; descriptive bibliometric approach
de Oliveira et al., 2021 [19]	Nursing professionals	Global bibliometric mapping	Nursing professionals only
Yang et al., 2024 [20]	Nurses	Publication trends, thematic evolution, citation analysis	Nursing-specific focus; descriptive bibliometric approach
Irigoyen-Amparan et al., 2025 [22]	Healthcare workforce	Publication trends, thematic evolution, collaboration networks	Descriptive bibliometric analysis; no multilevel citation modelling
Present study	Entire healthcare workforce	Science mapping, critical literature synthesis, multilevel citation modelling	Single WoS database; English-language restriction; search-strategy limitations; citation-window effects

**Table 2:** Information related to the articles included in the analysis.

Key Data Insights	
Time Frame	1987–2024
Sources	492
Number of documents	1232
Annual growth rate %	4.83
Average age of the document	4.7
Average number of citations per document	27.66
Number of references	39,338
Document Contents	
Keywords Plus (ID)	1414
Author keywords (DE)	2314
Authors	
Authors number	7035
Documents with a single author	46
Authors’ Collaboration	
Single-authored docs	48
Co-authors per Doc	6.55
International co-authors %	24.35
Types of Documents	
Articles	1047
Article: book chapter	5
Article: early access	22
Article: proceedings paper	6
Article: retracted publication	1
Review	150
Review: early access	1

**Table 3:** Multilevel Poisson and Negative Binomial Regression Models Predicting Citation Counts. Incidence Rate Ratios (IRRs) Are Additionally Reported for the Final Negative Binomial Model.

Variable		β Estimate		Std. Error		IRR		95% CI for IRR			z Value	*p* Value
(Intercept)		3.4025		0.2191		30.04		19.55–46.15			15.526	<0.001 ***
Authors_number		0.0044		0.0108		1.004		0.983–1.026			0.404	0.686
Abstract_length		0.0001		0.0006		1.000		0.999–1.001			0.260	0.795
Pages_number		0.0037		0.0059		1.004		0.992–1.015			0.626	0.531
References_number		0.0009		0.0021		1.001		0.997–1.005			0.421	0.674
Title_length		−0.0372		0.0090		0.963		0.947–0.981			−4.154	<0.001 *
Keywords_number		−0.0444		0.0217		0.957		0.917–0.998			−2.046	0.041 *
Keywords_plus_number		0.0747		0.0150		1.078		1.046–1.110			4.987	<0.001 *
**Statistic**										**Value**		
AIC										9361.9		
BIC										9411.6		
logLik										−4670.9		
−2 × log(L)										9341.9		
Residual df										1052		
Random-effect variance										0.6351		
Random-effect SD										0.7969		
Dispersion parameter										0.751		
**Model**	**AIC**		**BIC**		**LogLik**		**Deviance**		**Model Comparison**			
Poisson	43,582.5		43,627.3		−21,782.3		43,564.5		—			
Negative binomial	9361.9		9411.6		−4670.9		9341.9		LR χ^2^(1) = 34,223, *p* < 2.2 × 10^−16^			

Note: The multilevel regression models were estimated using an analytical dataset comprising 1062 publications, whereas the descriptive bibliometric analyses were performed on the complete bibliometric dataset (*n* = 1232). Incidence Rate Ratios (IRRs) and their 95% confidence intervals were obtained by exponentiating the regression coefficients and their corresponding Wald confidence limits. An IRR > 1 indicates a positive association with the expected citation count, whereas an IRR < 1 indicates a negative association. Significance levels: * *p* < 0.05; *** *p* < 0.001.

## Data Availability

The complete Boolean search strategy is reported in the Methods section. Supplementary File S2 provides the coding dictionary describing the variables, professional classification criteria, and country classification rules used in the bibliometric and multilevel citation analyses. The bibliographic records were retrieved from the Web of Science Core Collection and are subject to the database licensing conditions. Additional documentation supporting the bibliometric procedures described in the manuscript is available from the corresponding author upon reasonable request, subject to the licensing restrictions applicable to the original Web of Science records.

## Supplementary Materials

The following supporting information can be downloaded at: https://www.mdpi.com/article/10.3390/healthcare14152356/s1, Table S1. Structured critical literature synthesis of the purposively selected studies [9,20,21,31,32,33,34,35,36,37,38,39,40,41,42,43,44,45,46,47]. Table S2: Descriptive statistics. File S2. Coding Dictionary for the Bibliometric and Multilevel Citation Analyses.
