# Supplementary material for: Global Trends, Inequalities, and Citation Dynamics in Burnout Research Among Healthcare Professionals: A Bibliometric Analysis (1987–2024)
Source: Healthcare (Basel). 2026 Aug 2;14(15):2356. doi: 10.3390/healthcare14152356 (PMC13464607; doi:10.3390/healthcare14152356)
Supplement: Supplementary file 1 [file healthcare-14-02356-s001.zip › healthcare-4425462-S2.pdf]

Supplementary File S2. Coding Dictionary for the Bibliometric and Multilevel Citation Analyses

This supplementary file describes the variables, coding procedures, and classification rules applied in the bibliometric and multilevel citation analyses. It is provided to improve the transparency and reproducibility of the methodological procedures reported in the manuscript.

Variable	Description	Coding / Measurement
Publication year	Year of publication indexed in the Web of Science Core Collection	Numeric (1987–2024)
Citation count	Total number of citations recorded in the Web of Science Core Collection at the time of data extraction	Numeric
Journal	Journal in which the article was published	Text
Document type	Publication type included in the study	Article; Review
Number of authors	Total number of authors listed for each publication	Numeric
Title length	Number of words in the article title	Numeric
Abstract length	Number of words in the abstract	Numeric
Author keywords	Number of author-provided keywords	Numeric
Keywords Plus	Number of Keywords Plus terms indexed in Web of Science	Numeric
Number of references	Total number of cited references	Numeric
Page count	Total number of pages	Numeric
Professional group	Principal healthcare professional population investigated in each publication	Physician; Nurse; Allied health professionals; Administrative personnel; Mixed healthcare professionals; Other healthcare professionals
Country	Country assigned according to the affiliation of the first author	Country name as indexed in Web of Science
Research theme	Main thematic area identified through bibliometric mapping	Derived from keyword co-occurrence and thematic analyses

Variable	Description	Coding / Measurement
Journal cluster	Journal-level grouping used in the multilevel negative binomial regression	Random effect

Professional Classification Rules

Publications were classified according to the principal healthcare professional group investigated in the study.

The following categories were applied:

- **Physicians** – studies primarily involving medical doctors.
- **Nurses** – studies focused predominantly on nursing personnel.
- **Allied health professionals** – pharmacists, physiotherapists, psychologists, occupational therapists, radiographers, laboratory personnel, paramedics, midwives, and other allied health professions.
- **Administrative personnel** – healthcare managers, administrators, and non-clinical administrative staff.
- **Mixed healthcare professionals** – studies including two or more professional groups without a single dominant occupational category.
- **Other healthcare professionals** – professional categories that could not be assigned to the previous groups.

When mixed professional populations were investigated and no single occupational group predominated, the publication was classified as **Mixed healthcare professionals**.

Country Classification Rules

Country assignment was based on the institutional affiliation of the **first author**, as indexed in the Web of Science Core Collection.

When authors represented multiple countries, only the country of the first author was used for country-level analyses to ensure consistency across publications.

Bibliographic Variables

Bibliographic characteristics (publication year, journal, document type, citation count, title length, abstract length, author keywords, Keywords Plus, number of references, and page count) were extracted directly from the bibliographic records retrieved from the Web of Science Core Collection and used without modification, except where numerical variables were summarized for descriptive or regression analyses.

Regression Variables

The multilevel negative binomial regression model included:

- Citation count (dependent variable)
- Title length
- Number of authors
- Abstract length
- Number of author keywords
- Number of Keywords Plus terms
- Number of references
- Page count

Journal was specified as a random intercept to account for clustering of publications within journals.

Notes

- Bibliographic records were retrieved exclusively from the **Web of Science Core Collection**.
- Professional classifications were assigned manually according to the predefined criteria described above.
- The coding framework was developed before the descriptive and regression analyses to ensure consistency throughout the study.
- This coding dictionary documents the variables and classification procedures used in the bibliometric and multilevel citation analyses reported in the manuscript.
