# Supplementary material for: Global Trends, Inequalities, and Citation Dynamics in Burnout Research Among Healthcare Professionals: A Bibliometric Analysis (1987–2024)
Source: Healthcare (Basel). 2026 Aug 2;14(15):2356. doi: 10.3390/healthcare14152356 (PMC13464607; doi:10.3390/healthcare14152356)
Supplement: Supplementary file 1 [file healthcare-14-02356-s001.zip › healthcare-4425462-supplementary.pdf]

Supplementary Material

Supplementary Table S1 supports the qualitative synthesis, whereas Supplementary Table S2 presents descriptive statistics for the variables included in the multilevel citation models.

Supplementary Table S1. Structured critical literature synthesis of the purposively selected studies.

Study	Dataset	Methodological approach	Challenges	Results	Limitations
1. Subarkah, F., and Iskandar, Y. (2024). A bibliometric review of factors causing burnout. Buletin Poltanesa 25(1), 31–37. doi:10.51967/tanesa. v25i1.3041. [31]	Publications on burnout retrieved from PubMed, Scopus, and Web of Science covering approximately the last decade.	- Bibliometric analysis using VOSviewer to identify publication trends, research themes, and influential contributors.	- Limited understanding of the specific causal factors contributing to burnout and insufficient evidence to guide effective prevention strategies.	Burnout research has expanded substantially, with excessive workload, work–life imbalance, and inadequate social support identified as major contributing factors.	Few studies specifically examined burnout determinants, highlighting the need for further research on causal mechanisms.
2. Yang, Z., Zhang, M., Guo, Y., Wang, R., and Xie, F. (2024). Burnout among nurses: A bibliometric analysis of the global publications. Psychol. Res. Behav. Manag. 17, 1727–1739. doi:10.2147/prbm.s458 199. [20]	Web of Science publications on nurse burnout published between 2003 and 2022.	Bibliometric analysis using bibliometrix and VOSviewer to examine publication trends, collaboration networks, and research hotspots.	Increasing burnout among nurses worldwide and the need to identify major research themes and emerging trends in the field.	Scientific output on nurse burnout increased substantially over time, with workload, emotional exhaustion, mental health, and COVID-19 emerging as major research themes. International collaboration and interdisciplinary research also increased.	The analysis was restricted to Web of Science records and may not fully represent studies indexed in other databases.

3.Sriharan, A., Ratnapalan, S., Tricco, A.C., and Lupea, D. (2021). Women in healthcare experiencing occupational stress and burnout during COVID-19: A rapid review. BMJ Open 11(4), e048861. doi:10.1136/bmjopen-2021-048861. [32]	Literature identified from MEDLINE, Embase, CINAHL, PsycINFO, and ERIC covering December 2019 to September 2020; 47 studies were included in the final review.	Rapid review examining occupational stress and burnout among women healthcare professionals during the COVID-19 pandemic.	Major stressors included safety concerns, workload, staff and resource shortages, and job insecurity during the pandemic.	Safety concerns were the most frequently reported stressor. The review identified substantial occupational stress among women healthcare workers and found limited evidence supporting organizational interventions compared with individual coping strategies.	Most included studies were observational and focused on early pandemic experiences, limiting long-term conclusions
4. Sweileh, W.M. (2020). Research trends and scientific analysis of publications on burnout and compassion fatigue among healthcare providers. J. Occup. Med. Toxicol. 15(1), 1–10. doi:10.1186/s12995-020-00274-z. [21]	Scopus publications on burnout and compassion fatigue among healthcare providers published up to 2019 (n = 4,416).	Bibliometric analysis of publication trends, research themes, citations, and leading contributors using the Scopus database.	Limited evidence from low- and middle-income countries and potential underrepresentation of non-indexed journals.	Publications increased substantially over the last decade, with nurses and physicians accounting for most studies. The United States was the leading contributor, highlighting significant geographic inequalities in research output.	Findings were restricted to Scopus-indexed publications and may not fully reflect research activity in underrepresented regions.
5. Tawfik, D.S., Scheid, A., Profit, J., Shanafelt, T.D., Trockel, M., Adair, K.C., Sexton, J.B., and Ioannidis,	Publications retrieved from MEDLINE, PsycINFO, Embase, Web of Science, and related databases	Systematic review and meta-analysis conducted according to PRISMA and MOOSE guidelines to examine	High heterogeneity across studies and potential publication bias complicated the interpretation of the	Burnout was frequently associated with poorer quality of care, patient safety concerns, and medical	Most included studies were observational, preventing causal inference, and substantial

J.P.A. (2019). Evidence relating health care provider burnout and quality of care: A systematic review and meta-analysis. Ann. Intern. Med. 171(8), 555–567. doi:10.7326/M19-1152. [33]	through May 2019; 123 studies were included in the review and meta-analysis.	associations between healthcare provider burnout and quality of care outcomes.	relationship between burnout and quality of care.	errors. Emotional exhaustion showed the strongest and most consistent associations with adverse quality outcomes.	heterogeneity limited the precision of pooled estimates.
6. Soares, J.P., Lopes, R.H., Mendonça, P.B.S., Rodrigues, C.C.F.M., and de Castro, J.L. (2022). Use of the Maslach Burnout Inventory among public health care professionals: A scoping review. JMIR Ment. Health 10, e44195. doi:10.2196/44195. [9]	Studies identified from six databases and gray literature sources; 55 articles published between 1999 and 2021 were included.	Scoping review conducted according to Joanna Briggs Institute methodology and PRISMA-ScR guidelines to examine the use of the Maslach Burnout Inventory among healthcare professionals.	Variability in the application and interpretation of the Maslach Burnout Inventory complicated comparisons across studies and healthcare settings.	Most studies focused on physicians and nurses and used different versions of the Maslach Burnout Inventory with varying scoring systems and cutoff values. The findings highlighted substantial heterogeneity in burnout measurement and classification.	Differences in MBI adaptations and the scoping review design limited comparability and prevented detailed evaluation of causal relationships.
7. Qin, Z., Yang, Q., Meng, Z., Lei, Q., Wen, J., Shi, X., Liu, J., and Wang, Z. (2023). Prevalence and correlators of burnout among health professionals during	Three online surveys of healthcare professionals conducted during different stages of the COVID-19 pandemic in China.	Repeated cross-sectional study using the MBI-HSMP, PHQ-9, and GAD-7 instruments to assess burnout, depression, and anxiety. Logistic regression was used to	Persistent burnout among healthcare professionals during the pandemic and the influence of workplace violence, workload, and demographic factors.	High levels of emotional exhaustion, reduced personal accomplishment, depression, and anxiety were observed across all pandemic phases. Workplace	Online self-reported surveys may be subject to response bias and do not allow causal inference.

different stages of the COVID-19 crisis in China. Front. Psychiatry 14, 1183060. doi:10.3389/fpsyt.2023. 1183060. [34]		identify associated factors.		violence and female gender were associated with higher	
8. Czapla, M., Milecka, D., Kowalczuk, K., and Uchmanowicz, I. (2023). Occupational burnout among nursing professionals: A comparative analysis of 1103 Polish female nurses across different hospital settings. Sustainability 15(11), 8628. doi:10.3390/su1511862 8. [35]	Data from 1,103 Polish female nurses working in surgical, intensive care, and non-invasive hospital units.	Cross-sectional study using the Maslach Burnout Inventory (MBI) and Mini-COPE questionnaire to assess burnout and coping strategies.	Occupational burnout associated with workload, staffing shortages, emotional demands, and workplace stress, with differences observed across clinical settings.	Nurses in intensive care and non-invasive units reported higher burnout levels than those in surgical wards. More effective coping strategies were associated with lower burnout levels.	-The study included only female nurses and was conducted during the COVID-19 pandemic, limiting generalizability.
9. Leiter, M.P. (2015). Key worklife areas contributing to health care burnout: Reflections on the ORCAB project. Br. J. Health Psychol. 20(2), 223–227. doi:10.1111/bjhp.12124. [36]	Qualitative evidence from focus groups involving healthcare professionals and patients across several European countries, supplemented by existing research on burnout and worklife factors.	Thematic analysis exploring key worklife factors associated with burnout and quality of care in healthcare settings.	-Excessive workload and conflicts between personal and organizational values were identified as major contributors to burnout and reduced work engagement.	Six worklife areas—workload, control, reward, community, fairness, and values—were identified as key determinants of burnout and employee wellbeing. Improvements in these domains may reduce	The framework may not fully capture all physical and emotional demands of healthcare work and was based primarily on qualitative evidence.

				burnout and enhance quality of care.	
10. Sun, H., Zhang, T., Wang, X., Wang, C., Zhang, M., and Song, H. (2023). The occupational burnout among medical staff with high workloads after the COVID-19 and its association with anxiety and depression. Front. Public Health 11, 1270634. doi:10.3389/fpubh.2023.1270634. [37]	Survey data from 1,505 medical staff members collected after the COVID-19 pandemic.	Cross-sectional study using the Maslach Burnout Inventory–Human Services Survey (MBI-HSS) and statistical analyses, including logistic regression, to identify factors associated with burnout.	High workloads, insufficient psychological support, poor health status, sleep deprivation, and work-related pressures contributed to occupational burnout.	High levels of emotional exhaustion, depersonalization, and reduced personal accomplishment were reported. Burnout was significantly associated with anxiety, depression, insufficient sleep, and adverse working conditions.	The cross-sectional design limited causal inference, and some potentially relevant workplace factors were not assessed.
11. Salas-Vallina, A., Herrera, J., and Rofcanin, Y. (2023). Human resource management, quality of patient care and burnout during the pandemic: A job demands-resources approach. Employee Relat. 45(5), 1203–1221. doi:10.1108/er-10-2022-0485. [38]	Data collected from 499 physicians working in specialized medical units during the COVID-19 pandemic.	Cross-sectional study using Partial Least Squares Structural Equation Modeling (PLS-SEM) to examine relationships between human resource management practices, burnout, and quality of patient care.	High job demands increased burnout risk and negatively affected physician wellbeing and quality of care.	Effective human resource management practices were associated with lower burnout levels and improved quality of patient care. Job demands and job resources acted as important mediating factors.	Findings were limited to the healthcare sector and may not be generalizable to other occupational settings.

12. McCarthy, C., and Bhandari, M. (2019). Cochrane in CORR®: Preventing occupational stress in healthcare workers. Clin. Orthop. Relat. Res. 477(5), 938–944. doi:10.1097/CORR.0000000000000735. [39]	Cochrane review including 58 intervention studies involving 7,188 healthcare workers.	Systematic review of randomized and controlled studies evaluating individual and organizational interventions to reduce occupational stress and burnout.	High burnout prevalence among healthcare workers and limited high-quality evidence regarding the effectiveness of intervention strategies.	Cognitive-behavioral training, relaxation techniques, and organizational interventions produced modest reductions in stress and burnout. The findings supported the potential value of both individual and workplace-based approaches.	Most studies were small and had a high risk of bias, limiting confidence in the reported intervention effects.
13. Konlan, K.D., Asampong, E., Dako-Gyeke, P., and Glozah, F.N. (2022). Burnout and allostatic load among health workers engaged in human resource-constrained hospitals in Accra, Ghana. BMC Health Serv. Res. 22(1), 1150. doi:10.1186/s12913-022-08539-5. [40]	Data from 1,264 clinical and non-clinical healthcare workers from three public hospitals in Accra, Ghana, including questionnaire responses and biological measurements.	Cross-sectional study using burnout questionnaires, resilience assessment, and biomarkers to examine the association between burnout and allostatic load.	Healthcare workers in resource-constrained settings faced chronic occupational stress related to workforce shortages, limited resources, and demanding working conditions.	Burnout prevalence was higher among non-clinical workers than clinicians. Burnout was strongly associated with increased allostatic load, indicating a link between occupational stress and physiological dysregulation.	The cross-sectional design prevented causal inference, and the measurement of allostatic load relied on experimental biomarker thresholds.
14. Hoopsick, R.A., and Las, S.G.M. (2023). Differential effects of healthcare worker burnout on	Cross-sectional survey data from 200 U.S. healthcare workers representing different occupational levels.	Logistic regression analyses were used to examine associations between burnout and psychotropic	Burnout may influence mental health outcomes and medication-related behaviors differently	Higher burnout levels were associated with changes in prescribed psychotropic medication among	Cross-sectional, self-reported data and non-probability sampling limited causal

psychotropic medication use and misuse by occupational level. Soc. Psychiatry Psychiatr. Epidemiol. 58, 1–11. doi:10.1007/s00127-023-02496-y. [41]		medication use or misuse, stratified by occupational group.	across healthcare occupations.	healthcare prescribers and administrators, while burnout was associated with increased psychotropic medication misuse among other healthcare workers.	inference and generalizability.
15. West, D., Krcmery, V., Szydlowski, S.J., Ramirez, B., and Costello, M.L. (2022). Preventing the burnout syndrome by creating a healthy and healing environment. Clin. Soc. Work J. 13(6), 76–79. doi:10.22359/cswhi_13_6_13. [42]	Not applicable (conceptual paper discussing burnout prevention strategies in healthcare organizations).	Narrative analysis of organizational and individual factors contributing to burnout and potential prevention strategies.	Burnout is driven by multiple organizational and individual factors, including workforce shortages, administrative burden, technological demands, and workplace stress.	The authors emphasized that reducing burnout requires systemic organizational changes, including supportive leadership, flexible work arrangements, wellbeing programs, and a positive workplace culture.	The paper was conceptual in nature and did not provide original empirical data to evaluate the effectiveness of the proposed strategies.
16. Rotenstein, L.S., Brown, R.L., Sinsky, C.A., and Linzer, M. (2023). The association of work overload with burnout and intent to leave the job across the healthcare workforce during COVID-19. J. Gen. Intern. Med. 38(8), 1920–1927.	Cross-sectional survey data from 43,026 healthcare workers across 206 large healthcare organizations during the COVID-19 pandemic.	Cross-sectional survey with propensity-weighted analyses examining associations between work overload, burnout, and intent to leave across healthcare occupations.	High levels of work overload and burnout threatened workforce wellbeing and retention across both clinical and non-clinical staff.	Burnout affected nearly half of respondents and was particularly common among nurses. Work overload was strongly associated with both burnout and intention to leave the job across occupational groups.	Voluntary survey participation may have introduced selection bias, and the study did not examine specific sources of work overload.

doi:10.1007/s11606-023-08153-z. [43]					
17. Wang, J., Wang, W., Laureys, S., and Di, H. (2020). Burnout syndrome in healthcare professionals who care for patients with prolonged disorders of consciousness: A cross-sectional survey. BMC Health Serv. Res. 20(1), 1–10. doi:10.1186/s12913-020-05694-5. [44]	Survey data from 93 healthcare professionals working with patients with prolonged disorders of consciousness in neuro-rehabilitation units in China.	Cross-sectional study using the Maslach Burnout Inventory–Human Services Survey (MBI-HSS) and demographic analyses to identify factors associated with burnout.	Healthcare professionals caring for patients with prolonged disorders of consciousness experienced high emotional demands and elevated burnout risk.	Burnout affected 65.6% of participants and was more common among nurses, younger professionals, unmarried staff, and those with fewer years of experience.	Small sample size and cross-sectional design limited generalizability and prevented causal inference.
18. Matsuo, T., Yoshioka, T., Okubo, R., Nagasaki, K., and Tabuchi, T. (2022). Burnout and its associated factors among healthcare workers and the general working population in Japan during the COVID-19 crisis: A nationwide cross-sectional internet-based study.	Nationwide internet survey including 12,650 Japanese workers, of whom 1,087 were healthcare workers.	Cross-sectional survey using the Mini-Z Burnout Scale and modified Poisson regression models to identify factors associated with burnout.	Healthcare workers experienced substantial burnout during the COVID-19 pandemic, particularly in the context of high workloads, insufficient workplace support, and resource constraints.	Burnout prevalence was high among both healthcare workers (33.5%) and the general workforce (31.0%). Long working hours and adverse personal or occupational circumstances were associated with increased burnout risk.	Internet-based sampling may limit generalizability, and the use of a single-item burnout measure may not capture all dimensions of burnout.

BMJ Open 12(11), e064716. doi:10.1136/bmjopen-2022-064716. [45]					
19. Zhou, T., Xu, C., Wang, C., Sha, S., Wang, Z., Zhou, Y., Zhang, X., Hu, D., Liu, Y., Tian, T.F., Liang, S., Zhou, L., and Wang, Q. (2022). Burnout and well-being of healthcare workers in the post-pandemic period of COVID-19: A perspective from the job demands-resources model. BMC Health Serv. Res. 22(1), 251. doi:10.1186/s12913-022-07608-z. [46]	Cross-sectional survey data from 3,477 healthcare workers employed in 22 hospitals in Beijing, China.	ANOVA and hierarchical multiple regression analyses were used to examine the effects of job stressors, social support, and organizational support on burnout and psychological wellbeing.	COVID-19-related job demands contributed to burnout, anxiety, and depression among healthcare workers.	Higher job stressors were associated with increased burnout and psychological distress, whereas social and organizational support were associated with better wellbeing and lower burnout levels.	The cross-sectional design limited causal inference, and the dataset was not publicly available.
20. Siddiqui, I.N., Gupta, J., Collett, G., McIntosh, I.M., Komodromos, C., Godec, T., Ng, S.M., Maniero, C., Antoniou, S., Khan, R., Kapil, V.S., Khanji, M.Y., and Gupta, A. (2023). Perceived workplace	Longitudinal survey data collected from healthcare professionals in the United Kingdom during two phases of the COVID-19 pandemic, including 744 follow-up respondents.	Cohort analysis using logistic and linear regression models to examine associations between perceived workplace support, burnout, mental health, and wellbeing over time.	Variations in workplace support and pandemic-related pressures affected healthcare workers' mental health and burnout risk.	Higher perceived workplace support was associated with lower risks of anxiety, insomnia, depression, emotional exhaustion, and depersonalization, as well as better mental wellbeing. Improvements in	Potential selection bias and the use of a non-validated workplace support measure may limit generalizability and measurement accuracy.

support and mental health, well-being and burnout among health care professionals during the COVID-19 crisis: A cohort analysis. CMAJ Open 11(1), E191–E200. [47]			perceived support over time were associated with improved mental health outcomes.

The studies presented were purposively selected to represent different professional groups, geographical regions, methodological approaches, and thematic areas relevant to burnout research among healthcare professionals.

Supplementary Table S2: Descriptive statistics

	**Authors_ number**	**Title_ length**	**Keywords_ number**	**Keywords_ plus_number**	**Abstract_ length**	**References_ number**	**Citations_ number**	**Pages_ number**
Mean	5.98	15.93	5.26	6.00	245.62	51.55	33.63	12.06
Median	5.00	16.00	5.00	6.00	240.50	47.00	8.00	10.00
Mode	4.00	13.00	5.00	10.00	250.00	55.00	.00	10.00
Std. Deviation	3.967	4.859	2.098	3.300	96.147	27.707	98.162	13.951
Variance	15.742	23.612	4.404	10.895	9244.279	767.712	9635.876	194.647
Skewness	2.866	.411	.380	-.221	4.282	3.209	12.405	16.363
Kurtosis	21.202	.375	1.920	-1.249	41.794	26.278	238.900	342.100
Minimum	1.00	3.00	1.00	.00	1.00	2.00	.00	3.00
Maximum	54.00	36.00	16.00	10.00	1443.00	402.00	2268.00	355.00
